# Selenium nanoparticles enhance metabolic and nutritional profile in *Phaseolus vulgaris*: comparative metabolomic and pathway analysis with selenium selenate

**DOI:** 10.1186/s12870-025-06097-6

**Published:** 2025-01-28

**Authors:** Asmaa Abdelsalam, Fatma Abd El Lateef Gharib, Arezue Boroujerdi, Nada Abouelhamd, Eman Zakaria Ahmed

**Affiliations:** 1 Botany and Microbiology Department, Faculty of Science, Helwan University, Helwan, 11795 Egypt; 2 Chemistry Department, Claflin University, Orangeburg, SC 29115 USA

**Keywords:** Metabolomics, HSQC, Chemometrics analysis, KEGG pathway, Beans, Legumes

## Abstract

Selenium is a beneficial element in agriculture, particularly for its potential to improve plant growth and stress tolerance at suitable concentrations. In this study, *Phaseolus vulgaris* was foliar-sprayed with selenium selenate (Se) or selenium nanoparticles (SeNP) at different concentrations during the vegetative stage; afterward, the seed yield was analyzed for metabolomics using ^1^H, *J*-resolved and HSQC NMR data, and NMR databases. A total of 47 metabolites were identified with sugars being the major chemical class. In the control sample, the most abundant sugar was stachyose (14.6 ± 0.8 mM). Among the identified alkaloids, the concentration of trigonelline was the highest (0.6 ± 0.08 mM). Chemometric and cluster analyses distinctly differentiated the control from the Se and SeNP-treated samples. Treatments with SeNP resulted in elevated concentrations of sugars, carboxylic acids, and sulfur-containing amino acids compared to control and Se treated samples. Conversely, betaine levels were higher in Se samples. The presence of Se and SeNP significantly decreased the levels of several aliphatic amino acids, e.g. alanine. The addition of 50 µM SeNP upregulated the levels of trigonelline and syringate by 2-fold and 1.75-fold, respectively, relative to the control. Pathway analysis indicated the most significantly altered pathways due to SeNP addition were arginine biosynthesis and nitrogen metabolism. The pathways influenced by Se addition were glyoxylate and dicarboxylate metabolism as well as glycine-serine and threonine metabolism. This study proved that SeNP are more efficient than Se in enhancing the metabolic profile of *Phaseolus vulgaris* which will have implications for agricultural practices, focusing on the sustainability and nutritional enhancement of crops.

**Supplementary Information:**

The online version contains supplementary material available at 10.1186/s12870-025-06097-6.

## Introduction

In recent years, the agricultural sector has witnessed an increasing demand for sustainable practices that not only increase crop yield but also improve the nutritional value of food products. The significance of assuring the well-being of both the environment and human health has prompted research into innovative approaches that promote plant growth, nutrient absorption, and crop yield. The application of essential trace elements, such as selenium in agriculture has received considerable attention due to its role in plant development, yield improvements and stress protection [37, 46, 74] as well as its benefits for human nutrition [42].

Selenium is an essential micronutrient for selenoprotein biosynthesis in human [9, 65].Selenium is also an essential micronutrient that plays a key role in maintaining health and preventing a variety of diseases in both humans and animals [88]. Selenium functions as a beneficial micronutrient at low concentrations, promoting growth and increasing stress tolerance in plants [9].

Selenium intake at certain concentrations can cause health issues in plants as well as in animals and humans [26, 72]. One new approach in the field of agriculture is the application of selenium nanoparticles. These nanoparticles play a function in plant adaptability against stress circumstances [19, 39]. Selenium nanoparticles act as plant fertilizers for raising seed germination rates and increasing crop yields and productivity. Their small size and higher surface area make them more accessible to plant roots and help to absorb selenium.

*Phaseolus vulgaris*, also known as the common bean, is a basic legume in many parts of the world. Different parts of the plant contain minerals, carbohydrates, proteins, and dietary fiber content [11, 70]. Moreover, *Phaseolus vulgaris* is known to possess bioactive metabolites like phytosterols, phenols, and vitamins that have significant industrial and therapeutic properties [11].

The utilization of Nuclear Magnetic Resonance (NMR) spectroscopy has been increasingly prominent in the field of plant metabolomics, providing a valuable means of investigating and analyzing the metabolic composition of plants [2]. This technique offers a non-invasive and extremely informative methodology for characterizing the metabolic profile of plants. NMR spectroscopy facilitates a complete examination of the primary and secondary metabolites present in plant tissues [24]. This analytical technique allows researchers to gain insights into the metabolic reactions of plants in response to environmental stressors and treatments [1].

The objective of this study was to investigate and compare the effects of foliar application of Se and SeNP, at varying concentrations, on the metabolic profile, detailed metabolomic composition, and pathway modifications in the yield seeds of *Phaseolus vulgaris.*

## Materials and methods

### Plant material

*Phaseolus vulgaris* seeds were obtained from the Horticulture Research Institute, Agricultural Research Centre, located in Giza, Egypt.

### Selenium nanoparticles synthesis

Selenium nanoparticles were synthesized according to (Abouelhamd et al. [3]).In brief, the aqueous solution of sodium selenate was prepared by dissolving 0.18909 g of sodium selenate in 100 ml of deionized water. To this, 1.5 g of ascorbic acid was added, and the solution was incubated at ambient temperature until the color changed from colorless to orange, indicating the reduction process. Subsequently, a volume of 10 ml of a 10% gum arabic solution was added into a 10 ml selenium nanoparticles solution under constant agitation. The gum arabic solution was made by dissolving 1 g of gum arabic in 10 ml of deionized water, followed by heating the solution to 100 °C while maintaining continuous stirring.

### Selenium nanoparticles characterization

#### UV–visible spectrophotometer

The UV-intense absorbance of the nanoparticle solution was evaluated using a UV-visible spectrophotometer (ACCULAB Spectrophotometer Model UVS-260 D, DeKalb, IL, USA) within the wavelength range of 100 to 500 nm.

#### Transmission electron microscopy (TEM)

Using TEM electron microscopy (JEM-2100, JEOL, Tokyo, Japan), the size and morphology of SeNP were investigated. Nanoparticles of selenium were deposited on carbon-coated TEM grids to prepare for TEM investigations. The film on the TEM grids was allowed to dry, and the excess solution was blotted off with blotting paper.

#### Dynamic light scattering analyses

The average particle size, size distribution, and zeta potential of SeNP were determined through the utilization of a Zetasizer Nano ZS particle analyzer (Malvern Instruments, Malvern Ltd, Worcestershire, UK) using dynamic light scattering (DLS) experiments. The DLS measurements were conducted in accordance with the specified experimental parameters. The dispersant used in this study has a dielectric constant of 78.5. The material under investigation has a refractive index of 1.30. The dispersion, in this case is water, has a refractive index of 1.33. The temperature during the experiment was maintained at 25 °C. The viscosity of the dispersant was measured to be 0.8872 cp. The count rate recorded during the experiment was 306.2 kcps per second. The measurement position was set at 5.50 mm. The material absorption was found to be 0.001. The zeta cell used for the experiment was a clear disposable cell.

### *Phaseolus vulgaris* growth and treatments

Seeds that exhibited uniformity in both size and color were used in the present investigation. The seeds were germinated in clay pots. Each pot, approximately 40 cm in diameter, was filled with 15 kg of clay loamy soil. This soil composition consists of clay, silt, and sand in a ratio of 55%, 26%, and 19% respectively along with calcium carbonate 5.3% and 1% organic matter, pH = 7.5. The pots were consistently watered with tap water at specific intervals based on prevailing weather conditions to maintain the soil moisture content at 70% of its field capacity. During the vegetative phase, the plants underwent foliar spraying with 500 ml of solutions containing different concentrations (1.0, 5.0, 10.0, and 50.0 µM) of selenate or SeNP at intervals of 3 and 4 weeks after seed cultivation. Freshly synthesized gum arabic-stabilized SeNP were used to minimize aggregation and ensure particle homogeneity, with the suspension thoroughly mixed and dispersed prior to application. The selenium content in the sodium selenate solution and selenium nanoparticle (SeNP) suspension was quantified using Microwave Plasma Atomic Emission Spectroscopy (MP-AES) (Agilent Technologies 4210 MP-AES). The measured selenium concentrations were subsequently utilized to prepare equivalent selenium doses for both treatments, ensuring comparability. To achieve this, the SeNP suspension and sodium selenate solution were diluted based on their respective selenium content to standardize the concentrations for foliar application. Consistency in application was maintained by using a uniform spray volume per pot to achieve comprehensive leaf coverage until the point of dripping.

The control group of plants was sprayed with distilled water. At the fruiting time, which occurred approximately three and a half months after the seeds were initially cultivated, the seeds were harvested in a random manner from the plants. Each treatment includes six pots, each containing five plants. Each pot was considered as a single replicate.

### NMR sample collection: metabolite extraction and data collection

The dry seeds obtained from each treatment were finely crushed into a powder, ensuring complete homogenization. A quantity of 20 mg was taken from each sample for the metabolite extraction. The extraction of metabolites was conducted as described by Kim et al. [48] using a fixed proportion of methanol, chloroform, and water at a ratio of 2:2:1.8. The upper hydrophilic layer was isolated from the extract, and subjected to vacuum drying using a centrivap (Labcono^®^, Kansas City, MO, USA) for a duration of 24 h. Subsequently, the hydrophilic extracts were resuspended in 620 µl of TMSP NMR buffer. This buffer solution had 1 mM of deuterated Trimethylsilylpropanoic acid (TMSP-d4), 100 mM of sodium phosphate buffer (pH 7.3), and 0.1% of sodium azide, dissolved in 99.9% D_2_O. Nuclear magnetic resonance (NMR) data was collected at 700 MHz (Bruker AvanceTM^III^). The NMR data was obtained with a spectrum width of 16.0 ppm and 64 K points, leading to an acquisition time of 2.9 s. During a 3-second recycling delay, on-resonance pre-saturation was employed to suppress the solvent. The initial increment of the presat-noesy spectra was acquired using a total of 120 scans, including 4 dummy scans, with a relaxation delay of 3 s and pre-saturation at the residual water frequency. The 90º pulse width for each sample was determined using the automatic pulse calculation experiment (pulsecal) in Topspin 3.5 software (Bruker BioSpin, Billerica, MA). The data collection process involved acquiring two-dimensional ^1^-^13^ C HSQC data using a Bruker hsqcedetgpsisp 2.2 pulse sequence. The proton (^1^H) resonance was detected in the F2 dimension, exhibiting a spectral width of 11 parts per million (ppm). Conversely, the carbon-13 (^13^C) resonance was identified in the F1 dimension, with a spectral width of 180 ppm.

### Metabolite identification, quantification and statistical analysis

The identification of metabolites was conducted by comparing the ^1^H data of the seed extracts with the data available in the Chenomx NMR Suite (Chenomx Inc. in Edmonton, Alberta, Canada). The ^1^-^13^ C HSQC data were cross-referenced with previously published ^1^-^13^ C HSQC data and data obtained from the Human Metabolome Database (HMDB) and Biological Magnetic Resonance Bank (BMRB) to validate the Chenomx assignments, particularly when dealing with overlapping peaks. Statistical analyses of the normalized NMR data were conducted using MetaboAnalyst 6.0 software (MetaboAnalyst 6.0 - a comprehensive server for metabolomic data analysis, https://www.metaboanalyst.ca/home.xhtml). The concentrations of each metabolite were measured by fitting the internal reference TMSP at a concentration of 1 mM in the sample spectra. Then, the discernible spectral peaks in the sample spectra were fitted using the 700 MHz standard compound spectra library of Chenomx. The principal component analysis (PCA) was adjusted to incorporate 95% confidence intervals. The construction of clustering analysis, specifically heat map and hierarchical cluster, was carried out utilizing Euclidean distance measurement and Ward clustering algorithms. The one-way analysis of variance (ANOVA) was adjusted to a significance level of *p* ≤ 0.05, and a post-hoc analysis using Tukey’s HSD (Honestly Significant Difference) method was conducted. Boxplots were constructed to visually represent the distribution of data for the 30 metabolites that were deemed most significant. These metabolites were selected based on their *p*-values, which were determined using ANOVA.

### KEGG pathway and enrichment analysis

Pathway analyses and enrichment analysis were utilized to visualize the metabolic pathways that were significantly affected by SeNP and Se treatments. A pathway study was carried out utilizing the scatter plot as a visualization tool, the Hypergeometric Test for enrichment analysis, and the Relative-between Centrality for topology measurement. KEGG-based metabolite sets were used for enrichment analysis and *Arachis hypogaea* (peanut) was utilized as the model organism.

## Results

### Characterization of selenium nanoparticles

#### Visual color

**Fig. 1:**
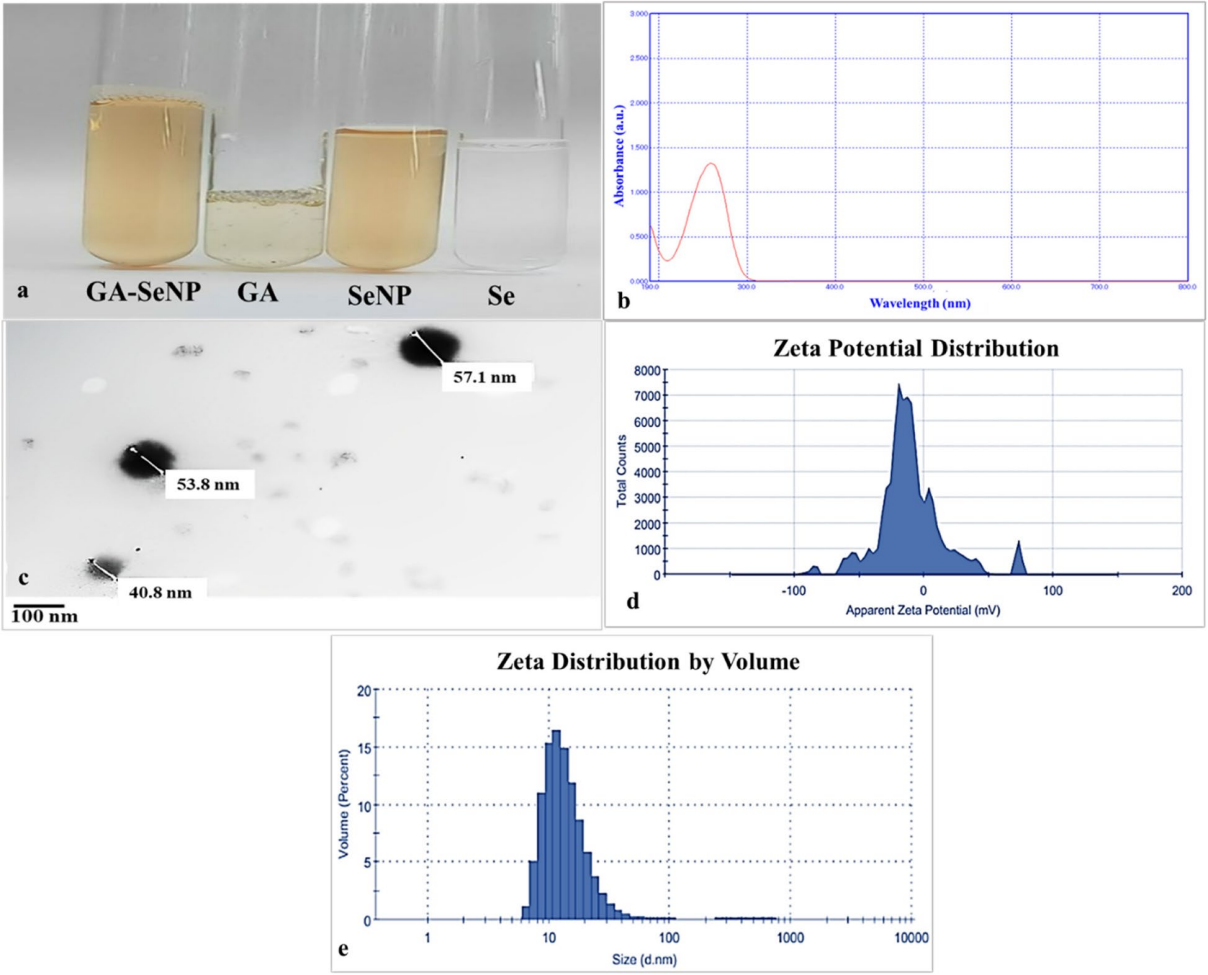
(**a**) SeNP coated with GA, GA solution, SeNP (without GA), and Se solutions, **b** UV-VIS spectra, **c** TEM photograph, and DLS measurements: **d** zeta potential and (**e**) size distribution of SeNP coated with GA

The synthesized SeNP exhibited a specific coloration when prepared using a solution containing 10 mM sodium selenate (Na_2_SeO_4_), 1.5% ascorbic acid at pH 2.6, and 10% gum arabic (Fig. 1a). The presence of a distinct bright orange color, serves as evidence for the successful synthesis of SeNP. This synthesis occurs through the reduction of selenium ions into nanoparticles by ascorbic acid.

#### UV–visible spectrophotometer

The ultraviolet spectrum of the SeNP solution was measured by ranging the wavelength from 190 to 800 nm with a resolution of 1 nm. The maximum absorption peak was detected at 270 nm (Fig. 1b).

#### Transmission electron microscopy (TEM)

The transmission electron microscopy (TEM) analysis revealed that SeNP exhibited a spherical morphology with an average diameter of around 46.9 nm (Fig. 1c).

#### Dynamic light scattering analyses

The DLS measurement (Fig. 1d & e) provided information on the zeta potential distribution of SeNP (with GA), which was determined to be −10.1 mV. The hydrodynamic diameter of SeNP (with GA) had an average value of 48.22 nm.

### Effect of selenium nanoparticles and selenium selenate on *Phaseolus vulgaris* seed weight

**Fig. 2:**
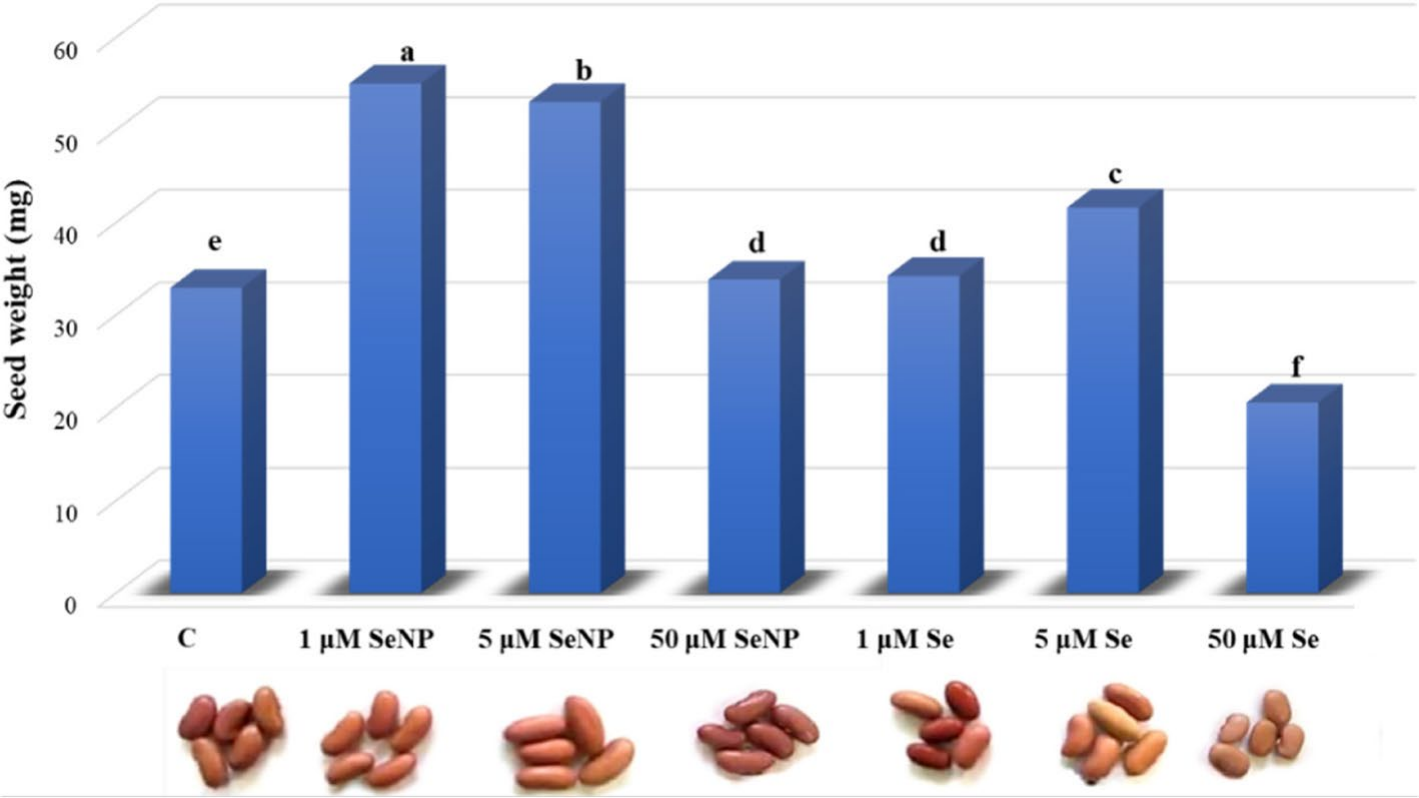
Effect of different concentrations of Se and SeNP on *Phaseolus vulgaris* seed dry weight (mg) and color. The different letters assigned to each column indicates the significance between mean of the groups being compared at *p* ≤ 0.05 level according to One-Way ANOVA

The impact of spraying *Phaseolus vulgaris* shoots with various concentrations of Se and SeNP on seed yield, morphology, and dry weight is shown in Fig. 2. The morphological variations observed in the seeds of *Phaseolus vulgaris* show that different doses of Se and SeNP have discernible impacts on their coloration. Lower concentrations of Se, specifically 1 µM have been observed to enhance the red coloration of the seed. Conversely, the seeds produced by plants exposed to the highest concentration of Se (50 µM) exhibited a noticeable lightening of the red color. SeNP exhibit a pronounced darkening effect, particularly at higher concentrations (50 µM). Regarding the seeds’ dry weights, the effect of SeNP and Se is dependent upon their concentration. Seed weight is significantly enhanced by low concentrations (1 µM and 5 µM) of both SeNP and Se, with the highest increase observed at 1 µM SeNP. Nevertheless, the application of a high concentration (50 µM) of Se results in a reduction in seed weight, whereas SeNP at the same concentration increase the seed weight, though with less efficacy.

### NMR based metabolic profiling of *Phaseolus vulgaris* seeds

**Fig. 3:**
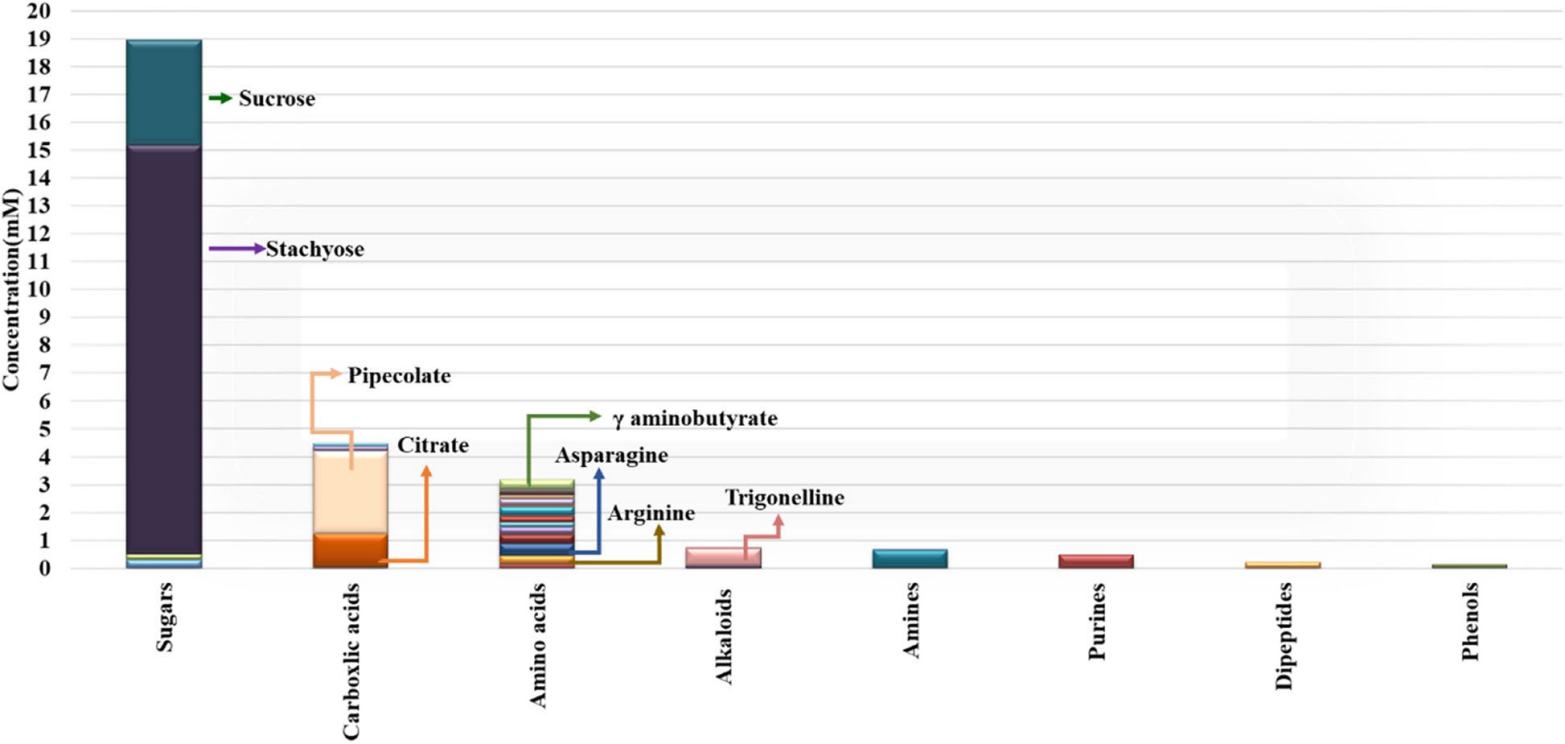
Concentration (mM) of the identified metabolites in the polar extract of control sample

**Fig. 4:**
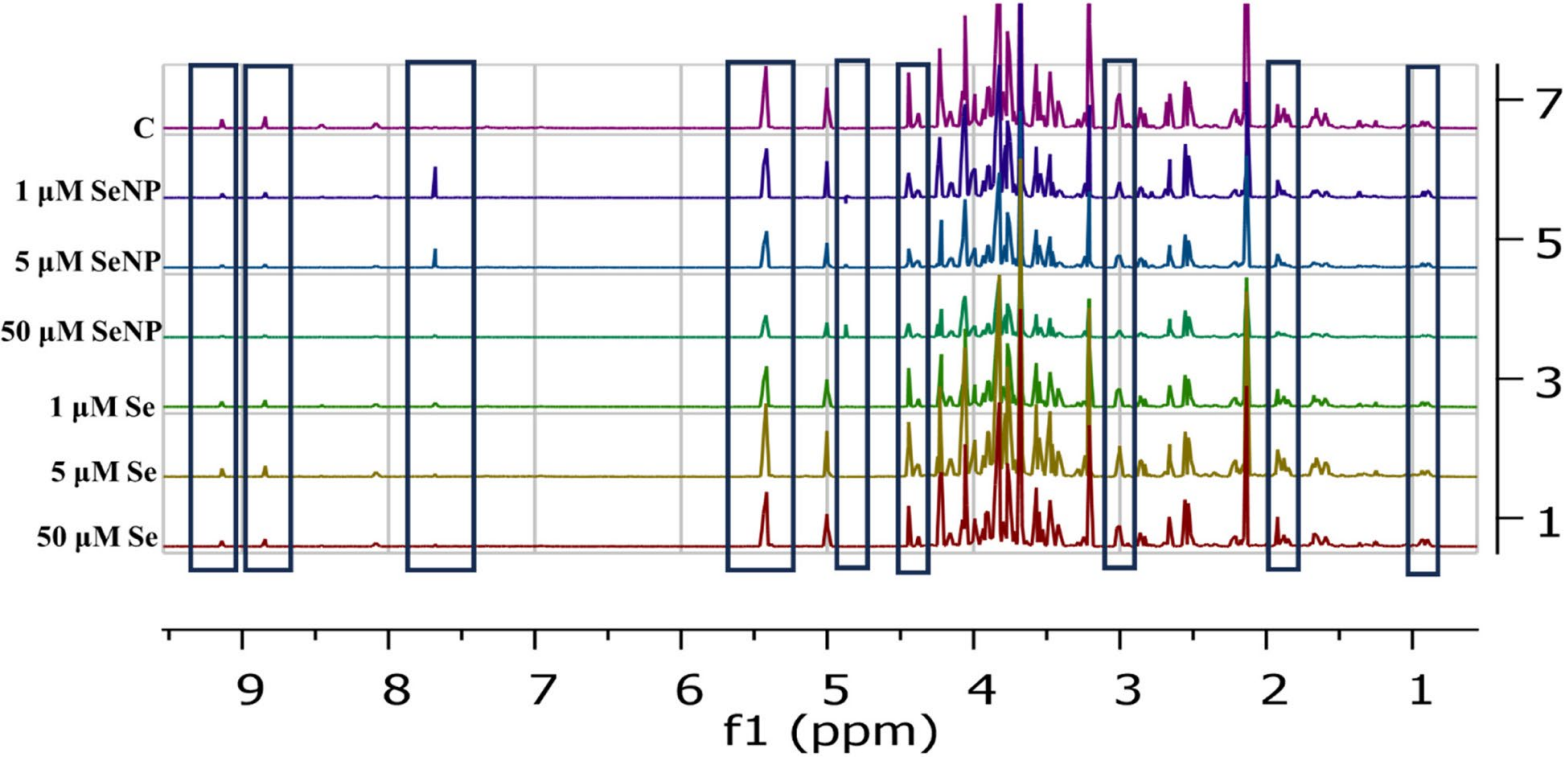
1D NMR stacked spectra for control (C), Se (1, 5, 50 µM) and SeNP (1, 5, 50 µM) treated samples

A total of 47 metabolites, belonging to various chemical classes, have been identified in seed extracts of control, SeNP, and Se treated samples. The identification was performed utilizing ^1^H and HSQC NMR data within the spectrum range of *δ* 0.5–10 ppm. The chemical formulas, molecular weights, *J*-resolved values, and ^1^H and ^13^C chemical shifts of the metabolites that have been identified are shown in Table 1. The concentrations of the identified metabolites in the control samples were determined by fitting a distinct and non-overlapping spectral characteristic of each compound with the corresponding standard spectrum provided by Chenomx. The seed extracts were composed of sugars, followed by carboxylic acids, amino acids, and alkaloids in descending order of concentration (Fig. 3). Four sugars have been successfully distinguished based on their unique signals in the spectral region (_*δ*_H 3.0–5.0 ppm). Among these sugars, stachyose has been identified as the major sugar, with a concentration of 14.6 ± 0.8 mM (Fig. 1, supplementary data). The most predominant amino acids found in the seed extract of the control sample are asparagine, γ aminobutyrate, aspartate, and arginine with concentrations of 0.44 ± 0.04 mM, 0.35 ± 0.06 mM, 0.31 ± 0.06 mM, and 0.30 ± 0.08 mM, respectively (Fig. 2 supplementary data). Pipecolate and citrate were major carboxylic acids with concentrations of 2.90 ± 0.2 and 1.23 ± 0.09 mM, respectively (Fig. 3 supplementary data). Various alkaloids were detected in the aromatic region (_δ_H 5.5–10 ppm), with trigonelline being the most abundant at a concentration of 0.6 ± 0.08 mM (Fig. 4 supplementary data).

The ^1^H NMR spectra of the control, SeNP-treated, and Se-treated samples exhibited distinct quantitative changes in the metabolites, as shown in Fig. 4.

**Table 1:** Metabolites identified in the polar extract of *Phaseolus vulgaris* seed polar extract

	Compound	Weight (g/mol)	Formula	^13^C chemical shift (ppm) (functional group or specific C)	^1^H chemical shift (ppm) (functional group or specific H, multiplicity of peak)	Coupling constant J (Hz)
1	2-Hydroxyisobutyrate	104.1045	C_4_H_8_O_3_	29.4 (CH_3_)	1.35(CH_3_, s)	-
2	2-Hydroxyisovalerate	118.1311	C_5_H_10_O_3_	18.2 (CH_3_) 21.2 (CH_3_)	0.82 (CH_3_, d) 0.95 (CH_3_, d)	4.02 7.04
3	γ-Aminobutyric acid	103.1198	C_4_H_9_NO_2_	26.4 (CH_2_) 37.0 (CH_2_) 42.0 (CH_2_)	1.89 (CH_2_, m) 2.29 (CH_2_, t) 3.01 (CH_2_, t)	- 7.3 7.4
4	Acetate	60.052	C_2_H_4_O_2_	26.1 (CH_3_)	1.90 (CH_3_, s)	-
5	Adenosine	267.2413	C_10_H_13_N_5_O_4_	155.2 (CH) 143.3 (CH)	8.21 (CH, s) 8.34 (CH, s)	8.9, 6.7 -
6	Alanine	89.0932	C_3_H_7_NO_2_	53.5 (C^α^) 18.9 (C^β^)	3.78 (H^α^, q) 1.47 (H^β^, d)	7.2, 7.2
7	Arginine	174.201	C_6_H_14_N_4_O_2_	26.6 (C^γ^) 30.3 (C^β^) 57.1 (C^α^)	1.64 (H^γ^, m) 1.93(H^β^, m) 3.77 (H^α^, t)	- - 6.2
8	Asparagine	132.1179	C_4_H_8_N_2_O_3_	54.2 (C^α^) 37.4 (C^β^)	4.00 (H^α^, q) 2.94 (H^β^, dd) 2.85 (H^β^, dd)	4.2 4.1, 16.9 7.7, 16.9
9	Aspartate	133.1027	C_4_H_7_NO_4_	54.9 (C^α^) 39.2 (C^β^)	3.89 (H^α^, dd) 2.67 (H^β^, dd)	8.80, 2.7 17.5, 8.8
10	Betaine	117.1463	C_5_H_11_NO_2_	69.2 (CH_2_) 56.2 (CH_3_)	3.89 (H^α^, s) 3.27 (H^β^, s)	- -
11	Choline	104.1708	C_5_H_14_NO	58.6 (C^α^) 56.8 (C^γ^) 70.4 (C^β^)	4.06 (H^α^_,_ m) 3.19 (H^γ^, s) 3.51 (H^β^, m)	- - -
12	Citrate	192.1235	C_6_H_8_O_7_	48.4 (CH_2_) 48.4 (CH_2_)	2.53 (CH_2,_ d) 2.66 (CH_2,_ d)	2.5 2.6
13	Formate	46.0254	CH_2_O_2_	-	8.44 (CH, s)	-
14	Fructose	180.1559	C_6_H_12_O_6_	78.2 (^3^CH) 66.1 (^6^CH_2_) 72.0 (^5^CH)	4.10 (^3^CH, dd) 4.01 (^6^CH_2_, dd) 3.99 (^5^CH, m)	12.7, 1.0 7.7, 5.4
15	Fumarate	116.0722	C_4_H_4_O_4_	138.0 (CH)	6.51 (CH, s)	-
16	Glycine	75.0666	C_2_H_5_NO_2_	44.3 (C^α^)	3.55 (H^α^, s)	-
17	Glucose	180.1559	C_6_H_12_O_6_	98.9 (^1α^CH) 94.8 (^1β^CH) 72.6 (^4^CH) 63.7 (^6^CH)	4.63 (^1α^CH, d) 5.22 (^1β^CH, d) 3.39 (^4^CH, m) 3.70 (^5β^CH, m)	7.8 3.6 - - -
18	Glutamate	147.1293	C_5_H_9_NO_4_	29.7 (C^β^) 36.2 (C^γ^) 57.3 (C^α^)	2.05 (H^β^, m) 2.35 (H^γ^, m) 3.75 (H^α^, dd)	- - 7.1, 4.7
19	Glutamine	146.1445	C_5_H_10_N_2_O_3_	33.7 (C^γ^) 29.1 (C^β^) 57.0 (C^α^)	2.43 (H^γ^, m) 2.14 (H^β^, m) 3.77 (H^α^, t)	- - 6.3
20	Glycyl-L-leucine	188.2242	C_8_H_16_N_2_O_3_	41.2 (CH_2_) 56.9 (CH)	1.58 (CH_2_, m) 4.19 (CH, s)	- -
21	Guanine	151.1261	C_5_H_5_N_5_O	149.0 (CH)	7.60 (CH, s)	-
22	Homoserine	119.1192	C_4_H_9_NO_3_	35.1 (C ^β^)	2.10 (H^β^, m)	-
23	Cysteine	121.1582	C_3_H_7_NO_2_S	28.8(C^β^) 57.2 (C^α^)	2.99 (H^β^, dd) 3.98(H^α^, dd)	
24	Isoleucine	131.1729	C_6_H_13_NO_2_	26.8 (C^γ^) 17.5 (C^γ^) 13.9 (C^δ^)	1.25 (H^γ^, m) 1.00 (H^γ^, d) 0.93 (H^δ^, t)	- 7.0 7.1
25	Leucine	131.1729	C_6_H_13_NO_2_	24.7 (C^δ^)	0.94 (H^δ^, t)	6.1
26	Malate	134.0874	C_4_H_6_O_5_	45.2 (CH_2_) 45.4 (CH_2_)	2.37 (CH_2_, dd) 2.66 (CH_2_, dd)	15.3, 10.2 15.3, 2.9
27	Methionine	149.2113	C_5_H_11_NO_2_S	56.8 (C^α^) 31.5 (C^γ^)	3.86 (H^α^, t) 2.63 (H^γ^, t)	6.2 6.6
28	N6-Acetyllysine	188.2242	C_8_H_16_N_2_O_3_	30.5 (CH_2_) 32.8 (CH_2_) 24.5 (CH_3_)	1.56 (CH_2_, m) 1.88 (CH_2_, m) 1.98 (CH_3_, s)	- - -
29	N-Acetyltyrosine	223.2252	C_11_H_13_NO_4_	24.6 (CH_3_) 39.5 (CH_2_) 39.4 (CH)	1.9 (CH_3_, s) 2.95 (CH_2_, m) 3.08 (CH, m)	- - -
30	N-Acetylornithine	174.1977	C_7_H_14_N_2_O_3_	32.4(CH_2_ ) 32.4(CH_2_ ) 24.5(CH_2_ )	1.72 (CH_2_, m) 1.85 (CH_2_, m) 2.05 (CH_3_, s)	- - -
31	O-Phosphocholine	184.1507	C_5_H_15_NO_4_P	56.5 (CH_3_) 86.9 (CH_2_) 60.6 (CH_2_)	3.22 (CH_3_, s) 3.58 (CH_2_, m) 4.16 (CH_2_, m)	- - -
32	Oxypurinol	152.1109	C_5_H_4_N_4_O_2_	128.8 (CH)	8.25 (CH, s)	-
33	Phenylalanine	165.1891	C_9_H_11_NO_2_	39.2 (C^β^) 58.9 (C^α^) 132.1 (C^δ^) 130.4 (C^ζ^) 31.8 (C^ε^)	3.12 (H^β^, dd) 3.99 (H^α^, dd) 7.31 (H^δ^, m) 7.37 (H^ζ^, m) 7.41 (H^ε^, m)	7.0 6.3 - - -
34	Proline	115.1305	C_5_H_9_NO_2_	64.2 (C^α^) 49.0 (C^δ^) 31.9 (C^β^)	4.13 (H^α^, m) 3.41 (H^δ^, m) 2.36 (H^β^, m)	- - -
35	Pipecolate	129.157	C_6_H_11_NO_2_	24.2 (CH_2_) 24.2 (CH_2_) 24.4 (CH_2_) 29.1 (CH_2_) 46.2 (CH_2_)	1.63 (CH_2_, m) 1.84 (CH_2_, m) 1.87 (CH_2_, m) 1.92 (CH_2_, m) 2.98 (CH_2_, ddd)	- - - - 12.4, 12.3, 2.6
36	Pyruvate	88.06	C_3_H_4_O_3_	29.3 (CH_3_)	2.36 (CH_3_, s)	-
37	Sarcosine	89.0932	C_3_H_7_NO_2_	35.6 (CH_3_) 53.4 (CH_2_)	2.72 (CH_3_, s) 3.60 (CH_2_, s)	- -
38	Stachyose	666.5777	C_24_H_42_O_21_	94.7 (CH) 69.0 (CH) 71.3 (CH) 79.0 (CH) 94.7 (CH)	3.49 (CH, m) 3.84 (CH, dd) 4.10 (CH, d) 4.22 (CH, dd) 5.40 (CH, m)	- 11.4, 5.4 11.4 11.4 -
39	Sucrose	342.2965	C_12_H_22_O_11_	95.1 (^1^CH) 79.8 (^3’^CH) 76.8 (^4’^CH) 73.9 (^2^CH) 72.1 (^3^CH) 65.3 (^6^CH_2_) 64.2 (^1’^CH_2_) 72.1 (^3^CH)	5.40 (^1^CH, d) 4.21 (^3’^CH, d) 4.04 (^4’^CH, t) 3.56 (^2^CH, m) 3.48 (^3^CH, m) 3.83 (^6^CH_2_, m) 3.67 (^1’^CH_2_, s) 3.46 (^3^CH, m)	3.89 8.8 38.6 - - - - -
40	Syringate	198.1727	C_9_H_10_O_5_	109.0 (CH) 58.7 (CH_3_)	7.26 (CH, d) 3.90 (CH_3_, s)	1.9 -
41	Succinate	118.088	C_4_H_6_O_4_	36.8 (CH_2_)	2.39 (CH_2_, s)	-
42	Threonine	119.1192	C_4_H_9_NO_3_	63.4 (C^α^) 23.0 (C^γ^)	3.58 (H^α^, d) 1.32 (H^γ^, d)	5.20 6.49
43	Trigonelline	137.136	C_7_H_7_NO_2_	148.2 (^2^CH) 147.4 (^4,6^CH) 130.5 (^5^CH) 51.1 (^1^CH_3_)	9.12 (^2^CH, s) 8.83 (^4,6^CH, t) 8.07 (^5^CH, t) 4.43 (^1^CH_3_, s)	8.80 7.37 - -
44	Tryptophan	204.2252	C_11_H_12_N_2_O_2_	121.1 (C^ζ3^) 124.8 (C ^η2^) 127.9 (C ^δ2^) 114.7 (C ^ς2^)	7.18 (H^ζ3^, m) 7.27 (H^η2^, m) 7.31 (H^δ2^, s) 7.52 (H^ς2^, ddd)	- - - 7.0, 1.4, 0.5
45	Uridine	244.2014	C_9_H_12_N_2_O_6_	72.0 (CH) 92.0 (CH)	4.21 (CH, dd) 5.90 (CH, d)	9.0, 4.3 9.0
46	Valine	117.1463	C_5_H_11_NO_2_	63.0 (C^α^) 20.8 (C^γ^) 19.5 (C^γ^)	3.60 (H^α^, d) 0.98 (H^γ^, d) 1.03 (H^γ^, d)	4.54 7.00 7.00
47	Xanthine	152.1109	C_5_H_4_N_4_O_2_	140.3 (CH)	7.90 (CH, s)	-

#### Effect of SeNP and Se concentrations on metabolomic analysis

**Fig. 5:**
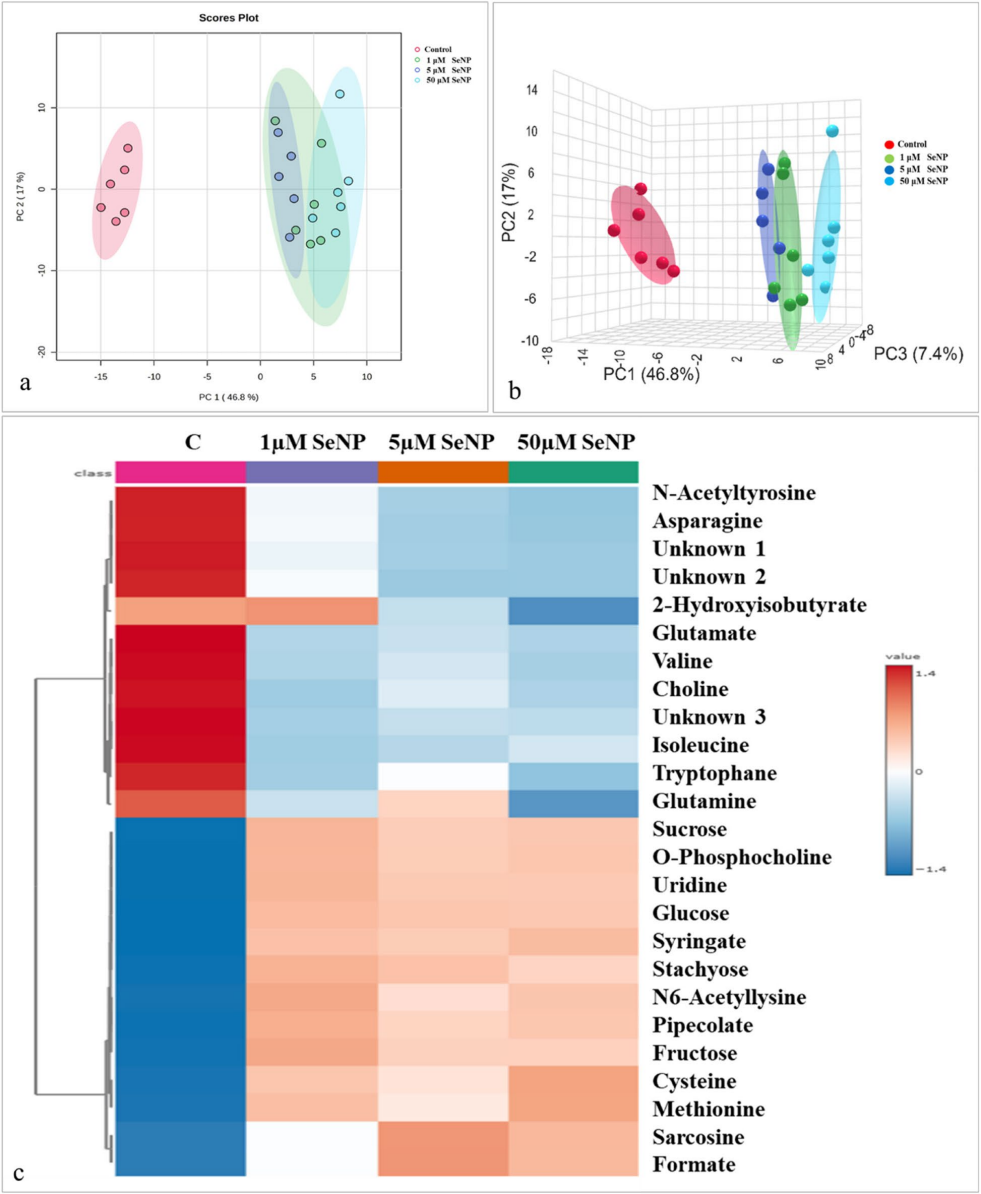
Principal component analysis (PCA) of SeNP. **a** 2D score plot, **b** 3D score plot. **c** Heat map dendrogram shows the metabolomic correlation between the control sample (**C**) and the samples treated with different SeNP concentrations. PCA was adjusted to incorporate 95% confidence intervals and the heat map was created using Euclidean distance measurement and Ward clustering algorithms. Each row represents a metabolite, and each column represents a sample

Chemometrics and cluster analysis were utilized to visually show the correlation between the polar extracts of control and SeNP-treated samples at the metabolomic level. The various concentrations of SeNP were observed to overlap in the 2D score plot, but were separated in the 3D score plot. The PCA score plots, both in 2D and 3D, demonstrated a distinct differentiation between the control samples and the samples treated with SeNP. The 2D and 3D score plots showed 63.8% and 71.2% of the total variation, respectively. The statistical evaluation of the PCA data was conducted using 95% Hotellings confidence intervals, as depicted in Fig. 5a & b.

The heat map analysis (Fig. 5c) illustrates the comparative concentrations of metabolites between the control samples and the different concentrations of SeNP-treated samples. The addition of SeNP resulted in a decrease in the levels of various aliphatic amino acids, such as aspartate, glutamate, valine, isoleucine, and tryptophan. In contrast, the addition of SeNP resulted in elevated levels of sulfur containing amino acids like cysteine and methionine, sugars like glucose, stachyose and sucrose, as well as the phenol compound syringate, along with organic acids like pipecolate and formate.

**Fig. 6:**
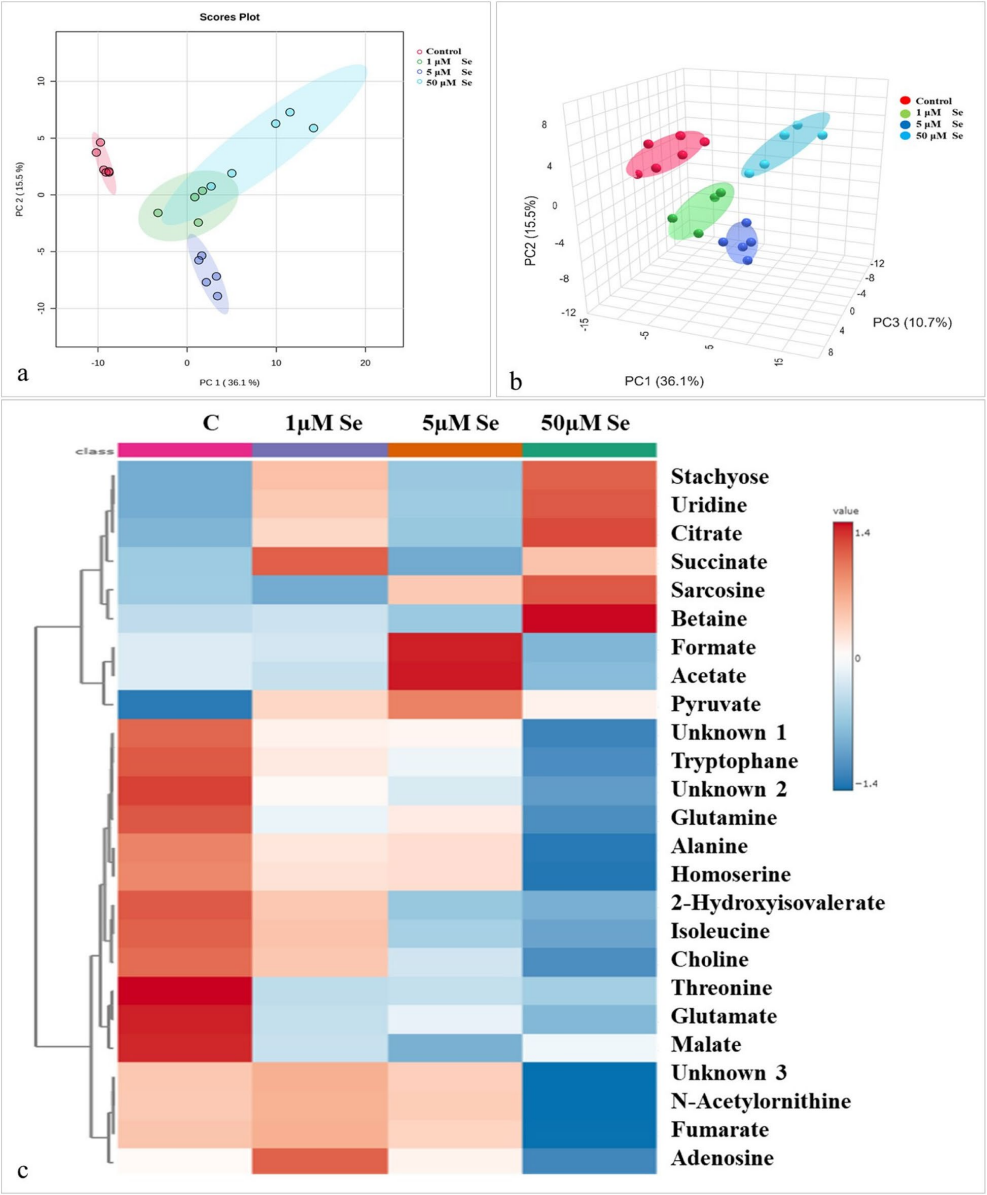
Principal component analysis (PCA) of Se. **a** 2D score plot, **b** 3D score plot. **c** Heat map dendrogram shows the metabolomic correlation between the control sample (**C**) and the samples treated with different Se concentrations. PCA was adjusted to incorporate 95% confidence intervals and the heat map was created using Euclidean distance measurement and Ward clustering algorithms. Each row represents a metabolite, and each column represents a sample

The PCA score plots, both in 2D and 3D (Fig. 6a & b), exhibited distinct separations between the control and Se treated samples. The score plot in both 2D and 3D showed total variances of 51.6% and 62.3%, respectively.

The heat map analysis (Fig. 6c) demonstrates that the addition of a high concentration of Se (50 µM) leads to a decrease in the levels of several amino acids, such as alanine, glutamine, and tryptophan, while causing a rise in the levels of betaine and sarcosine. The addition of 50 µM Se resulted in an increase in both citrate and succinate levels. On the other hand, addition of 5 µM Se increased the levels of formate.

**Fig. 7:**
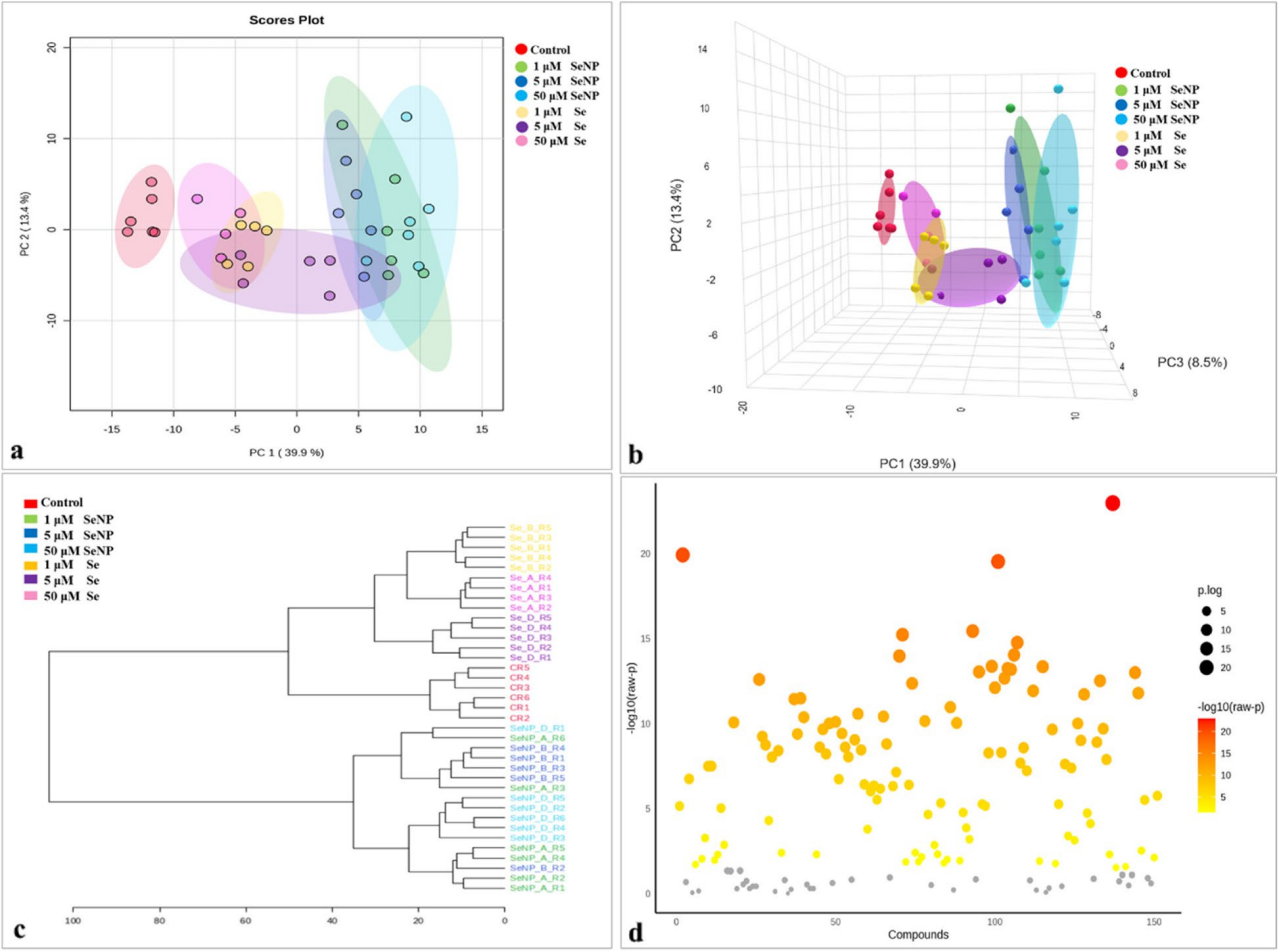
The effect of Se and SeNP on *Phasolus vulgaris* seeds: PCA score plots in (**a**) two and (**b**) three dimensions, **c** hierarchical cluster analysis (HCA), and (**d**) one-way analysis of variance (ANOVA). ANOVA was calculated using the 0.05 *p*-value threshold and (ANOVA) and a post-hoc analysis using Tukey’s HSD (Honestly Significant Difference)

PCA 2D and 3D score plots shown in Fig. 7a & b show separations between the control, Se and SeNP treated samples. The 2D and 3D score plots demonstrated 53.3% and 61.8% of the overall variance. The hierarchical clustering analysis (HCA) indicated the presence of two distinct main clusters. The control samples and Se treated samples were grouped together in the first cluster, which were further divided into two subclusters, one for control samples and the other for Se samples. The SeNP samples were grouped in the second cluster (Fig. 7c).

**Fig. 8:**
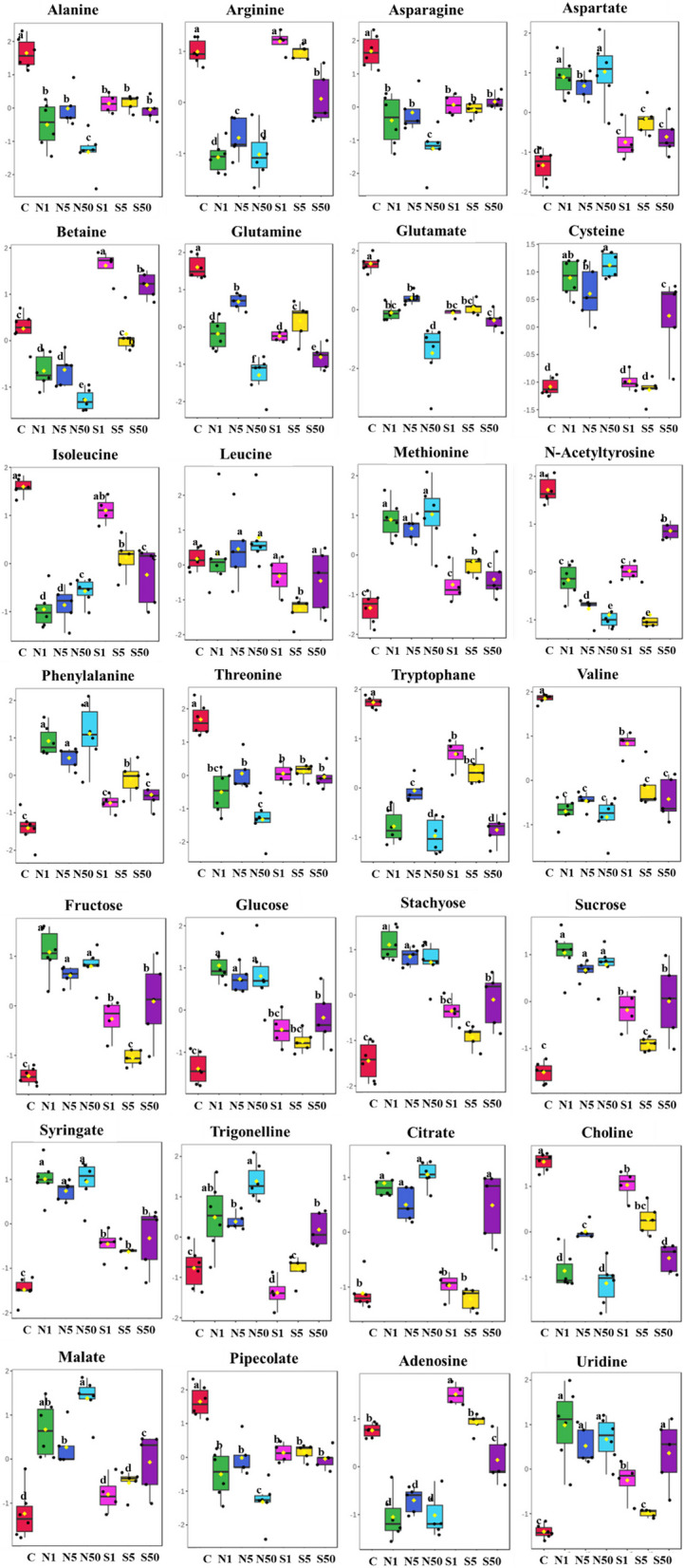
Boxplots of relative concentrations of the highest significant metabolites selected from ANOVA analysis that significantly changed between control (C), SeNP (N) with different concentrations (N1 = 1.0 µM, N5 = 5.0 µM, N50 = 50 µM, and Se (S) with concentrations (S1 = 1.0 µM, S5 = 5.0 µM, S50 = 50 µM). The black dots represent the concentration of the selected metabolite in each replicate. The notch indicates the 95% confidence interval around the median of each treatment, defined as +/- 1.58* IQR/sqrt (*n* = 6). The mean concentration of each treatment is indicated by the yellow diamond. The Y axis defines the relative abundances of the specific metabolite and the X axis defines the treatment group

A total of 114 metabolites exhibited significant alterations following the addition of different concentrations of SeNP and Se, as indicated by ANOVA presented in Fig. 7d. The box plots in Fig. 8 show the most prominent metabolites as determined by ANOVA. SeNP supplementation led to increased concentrations of some amino acids, including aspartate, cysteine, methionine, phenylalanine, and methionine, in comparison to both the control and Se-treated samples. Conversely, betaine was increased in samples treated with Se. The addition of SeNP at various concentrations reduced the amounts of many amino acids, such as alanine, arginine, isoleucine, and threonine.

All sugars exhibited upregulation upon the addition of SeNP and Se, as compared to the control group. Significantly, SeNP addition produced higher concentrations of sugars than Se.

The addition of SeNP at various concentrations resulted in the accumulation of alkaloids and phenols in the seeds of *Phaseolus vulgaris* than C and Se treated samples. The addition of 50 µM SeNP led to a significant increase of trigonelline, with 2-fold compared to the control. Additionally, the level of syringate was found to be 1.75 times higher than the control. In addition, the concentrations of citrate, malate, and pipecolate increased when SeNP was added.

### KEGG pathway and enrichment analysis

**Fig. 9:**
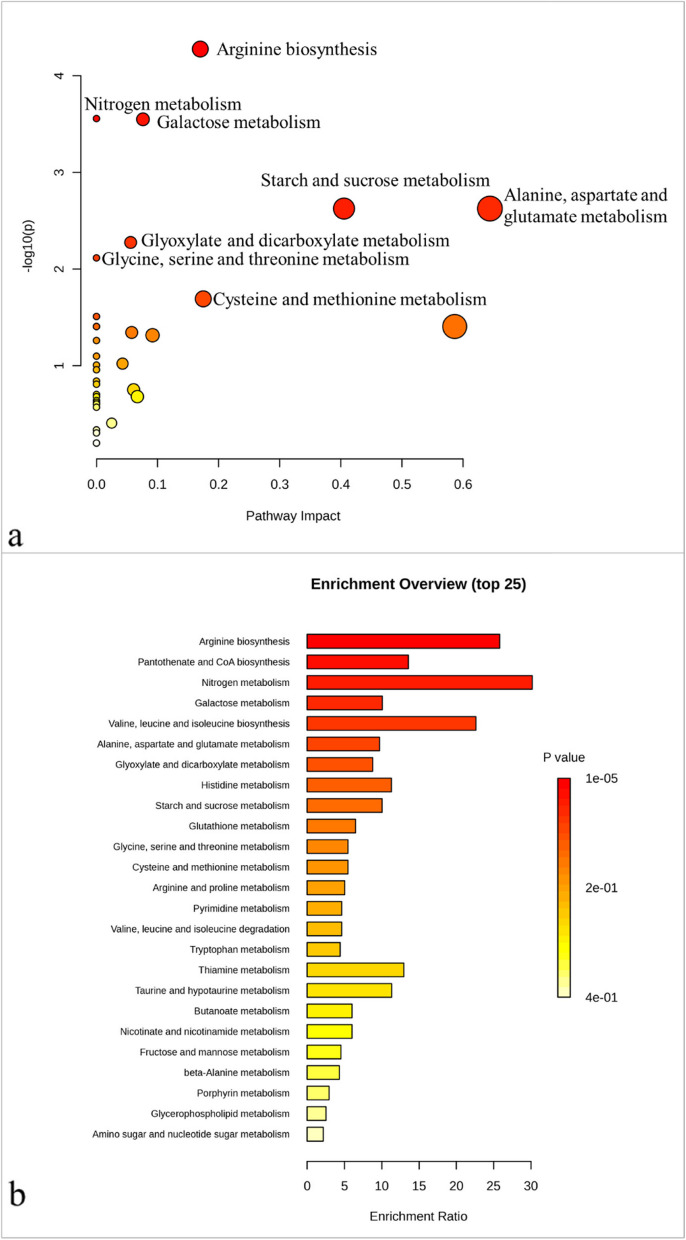
Metabolic pathways in *Phaseolus vulgaris* polar extract that are significantly altered with SeNP addition. **a** Pathway analysis (based on KEGG database) showing significantly changed metabolic pathways. The dark red circles indicate the pathways that were strongly affected by stress. As the *p*-value increases, the color progressively fades, whereas the larger the circle size, the greater the pathway’s influence. **b** Interactive bar-chart of the enrichment analysis (based on KEGG database). The most significant *p*-values are represented by dark red bars, while the color decreases gradually with decreasing *p*-values, with pale yellow representing the least significant; the length of the bar represents the enrichment ratio

Pathway analysis (Fig. 9a) revealed that the addition of SeNP had a significant effect on 34 metabolic pathways. The pathways that underwent the most significant alterations were arginine biosynthesis, nitrogen metabolism, and galactose metabolism, with *p* values of 0.00005, 0.00027, and 0.00028, respectively (Table 1 in the supplementary data). Meanwhile, the pathways most impacted were alanine, aspartate, and glutamate metabolism, as well as starch and sucrose metabolism. Enrichment analysis (Fig. 9b; Table 2 in the supplementary data) shows that the most important pathways that changed were those related to arginine metabolism, pantothenate and CoA biosynthesis, and nitrogen metabolism. The pathways that were most impacted were nitrogen metabolism, arginine biosynthesis, and valine, leucine, and isoleucine biosynthesis.

**Fig. 10:**
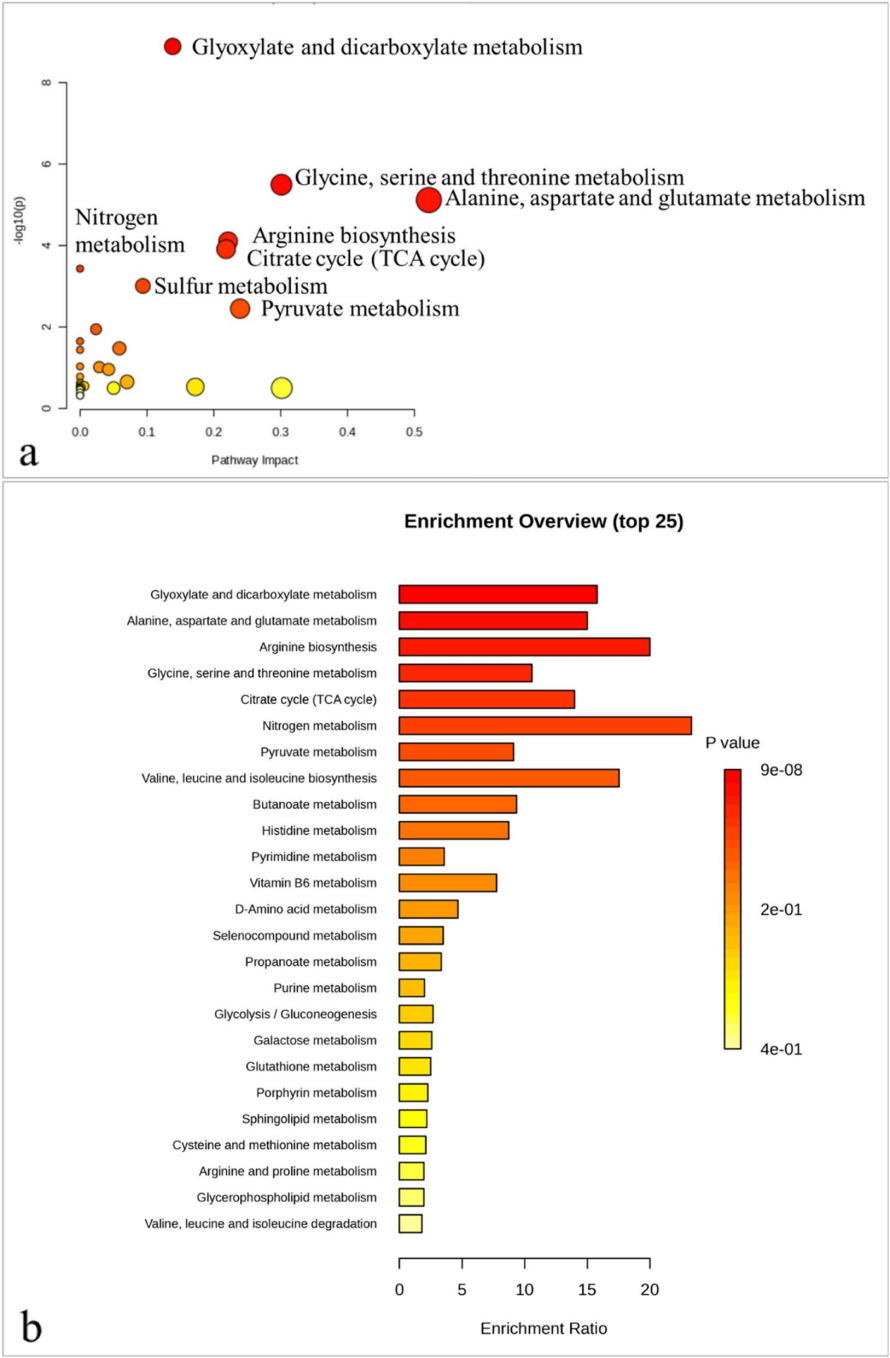
Metabolic pathways in *Phaseolus vulgaris* polar extract that are significantly altered with Se addition. **a** Pathway analysis (based on KEGG database) showing significantly changed metabolic pathways. The dark red circles indicate the pathways that were strongly affected by stress. As the *p*-value increases, the color progressively fades, whereas the larger the circle size, the greater the pathway’s influence. **b** Interactive bar-chart of the enrichment analysis (based on KEGG database). The most significant *p*-values are represented by dark red bars, while the color decreases gradually with decreasing *p*-values, with pale yellow representing the least significant; the length of the bar represents the enrichment ratio

The pathway analysis (Fig. 10a) explains that the addition of Se had a significant impact on 31 biological pathways (Table 3 in the supplementary data). The most significantly changed pathways were glyoxylate and dicarboxylate metabolism; glycine-serine and threonine metabolism; and alanine, aspartate, and glutamate metabolism. The alanine, aspartate, and glutamate metabolism pathways, as well as the glycine-serine and threonine metabolism pathways, had the greatest impact. The enrichment analysis (Fig. 10b; Table 4 in the supplementary data) reveals that glyoxylate and dicarboxylate metabolism, as well as alanine, aspartate, and glutamate metabolism, were the most significant pathways, while nitrogen metabolism and arginine biosynthesis pathways were the most impacted.

## Discussion

### Characterization of selenium nanoparticles

The present study describes the successful synthesis of selenium nanoparticles coated with gum arabic and their effects on the metabolic profile of *Phaseolus vulgaris* seeds. SeNP were effectively synthesized by a simple and low-cost chemical reduction procedure using sodium selenate, ascorbic acid, and gum arabic solution, which was used as a nanoparticle stabilizer. Ascorbic acid is one of the most used reducing agents in chemical synthesis of SeNP [14, 25]. Due to the high instability of pure SeNP in an aqueous solution, chemical reagents have a decisive role in their stabilization in contact with biological objects [86]. Gum arabic has been cited in multiple studies as a stabilizing agent for SeNP [50, 80].

The orange color of gum arabic-coated selenium nanoparticles indicates that the synthesis was done successfully. The color is similar to the one observed in previous studies that prepared selenium nanoparticles (without GA), and showed an orange color [41]. This color comes from the surface plasmon resonance of the particles, which imparts typical optical properties to nanoparticles; thus, it is a function of particle size and shape and can be further fine-tuned by the surrounding medium [7, 43]. Additionally, one remarkable absorption peak at 270 nm was obtained from the UV–Vis spectra of SeNP (with GA). This peak is comparable to the absorption characteristics reported by [17] and similar peaks identified in the region of 200–300 nm for selenium nanoparticles (without GA). The peak thus signifies the successful production of SeNP coated with gum arabic.

The DLS analysis provided insights into the size distribution and stability of SeNP (with GA). The average zeta potential is −10.1 mV. According to some interpretations, particles with zeta potentials ranging from − 10 mV to −30 mV are relatively stable; that is, their aggregation is slowed by significant electrostatic repulsion [12]. This means that nanoparticles are still regarded as colloidally stable. Higher absolute zeta potential values (more than ± 30 mV) provide more stable nanomaterials, whereas moderate stability in this range may be sufficient to prevent aggregate breakup in some applications. Nanoparticle stabilization ensures equal distribution and prevents agglomeration, making them suitable for use in medicines, cosmetics, and industrial processes [27]. The DLS measurement revealed a size diameter of 48.22 nm, which is consistent with the TEM investigation, which found an average diameter of 46.9 nm for SeNP (with GA). This uniformity reflects a restricted size distribution and the successful coating of selenium nanoparticles with gum arabic, which improves their stability and biocompatibility. TEM investigation confirmed the spherical form and well-aggregated character of SeNP (with GA), which is consistent with Vu et al. [80].

### Effect of Se and SeNP on seed weight

The increase in seed dry weight at low concentrations (1 µM and 5 µM) of Se is consistent with the findings of Rady et al. [64] who observed low concentrations of Se enhanced growth and yield in *Phaseolus vulgaris* plants. High S concentrations (50 µM) diminish seed weight, and were similarly reported by Tan et al. [76] on pea sprout. This is because a high concentration of Se in the plant distorts selenoproteins and induces oxidative stress [28]. On the other hand, SeNP have been shown to significantly increase seed dry weight across various concentrations. Lower concentrations of SeNP (1 µM and 5 µM) resulted in the most significant increases in seed dry weight. The positive effect of SeNP on the improvement of plant growth parameters has been previously reported [38]. Seed weight serves as an indicator of the average size of seeds [34], which is a crucial factor in assessing the yield of cultivated plant species.

### Metabolic profiling of *Phaseolus vulgaris* seed

The seed extracts are mostly composed of sugars, with stachyose being the most abundant. Similar findings were reported by [62]. Stachyose is an oligosaccharide with well-documented antimicrobial activity (Xi et al. [85]). The principal carboxylic acids found in the seed extracts were pipecolic acid and citric acid. Previous research identified pipecolic acid in bean [33], which is a by-product of lysine metabolism. This compound plays a crucial role in plant resistance [15, 49]. The most predominant essential amino acids were methionine, valine and tryptophan. Our findings are partially consistent with those of Hernández-Guerrero et al. [33] who found that methionine, tryptophan, and valine were the most abundant essential amino acids in *Phaseolus vulgaris* seed. Several alkaloids were found in the aromatic region with trigonelline being the most common. Trigonelline is a bioactive compound with anticancer, antimicrobial, anti-inflammatory and antioxidant activities [59]. Trigonelline has been previously reported in *Phaseolus vulgaris* extracts [33, 71].

### The effect of Se and SeNP on seed metabolomics

The use of chemometrics and cluster analysis showed clear differentiation between the SeNP-treated samples, Se-treated samples, and control samples. This distinctive clustering and high percentage of explained variance reflect the morphological differentiation of the seeds treated with SeNP and Se, indicating profound metabolic changes. It is worth mentioning that the samples treated with Se showed a metabolic similarity to the control samples, rather than the samples treated with SeNP. There are several factors that contribute to the greater impact of SeNP compared to Se. These factors include the fact that SeNP possess a nanoscale size, typically ranging from 1 to 100 nm, resulting in a substantial enhancement of their surface area-to-volume ratio. This property improves the interaction with plant surfaces, making absorption and translocation within the plant system more efficient [23, 26].

The heat map analysis demonstrates that the addition of Se at different concentrations has diverse impacts on the metabolite profile in plants, indicating that metabolic adjustments and stress responses are depending on the quantity of selenium. At a concentration of 50 µM of Se, there is a significant rise in the levels of betaine and sarcosine and decrease in choline concentration. Both betaine and sarcosine are substances that protect cells from osmotic stress [5, 52]. The increase in betaine levels may indicate an adaptive reaction to counter the oxidative or osmotic stress caused by elevated selenium levels. Choline serves as the precursor for betaine in various plant species [75], suggesting that a decrease in choline levels may be associated with its conversion to betaine, especially at higher selenium concentrations.

Based on box plot data, SeNP treatments showed high concentrations of the amino acids cysteine and methionine. Cysteine and methionine are sulfur containing, which are present in low concentrations in legume [4]. An increase in the concentrations of methionine, an essential amino acid required by humans, improves the nutritional value of legume seeds [30, 51]. Cysteine serves as a metabolic precursor for key molecules in plants, including antioxidants, vitamins, and enzyme cofactors [6]. It also plays a critical role in the plant immune defense against pathogens [67].

Sugars (glucose, fructose, and stachyose) were found to be upregulated with the addition of SeNP and Se. Sugars are essential for the growth of plants as they serve as the primary source of energy. The growth and development of plants depend on the control of sugar production and breakdown, as well as the influence of photosynthetic products and climatic conditions. The effect of Se and SeNP in enhancing sugar accumulation has been reported in various plant species. Selenium has been proven to regulate the sugar metabolism of rice [55]. Zhu et al. [91] proved that applying selenium at the initial growth stage of *Vitis vinifera* resulted in higher levels of fructose, glucose, sucrose. This finding is in line with the results obtained in the current investigation. Du et al. [22] reported that certain levels of selenium led to an increase in the amount of soluble carbohydrates and effect on α-amylase activity in rice. Selenium supplementation in *Brassica juncea* L. seedlings resulted in an elevation of sugar levels, as reported by Handa et al. [31].

The addition of SeNP at varying concentrations led to an increased accumulation of various bioactive metabolites, such as alkaloids and phenols, in comparison to the control and Se-treated samples. SeNP, with their distinct nano-properties, have the potential to affect secondary metabolism in plants in a different manner compared to traditional selenium treatments. These changes suggested that SeNP not only improve the plant’s stress tolerance and growth but also enrich its nutritional profile, making it potentially more beneficial for human consumption. SeNP treatment at a concentration of 50 µM resulted in a marked increase in the levels of trigonelline. Trigonelline is well-known for its health benefits [60]: antidiabetic, neuroprotective, anti-Alzheimer, and anticancer properties [56]. Also, it prevents oxidative stress in humans caused by ultraviolet-B [77]. Seeds of coffee and members of the Fabaceae family contain significant amounts of trigonelline, while smaller quantities can be found in various other species [8]. In legume plants, trigonelline has been reported as a signal for establishing a symbiotic relationship with *Rhizobium meliloti* bacteria [61]. Trigonelline has been reported to improve the plant tolerance to stress conditions [54].

The increased level of syringate in SeNP-treated samples compared to the control and Se-treated samples further contributes to the nutritional improvement of the seeds. Syringate is a phenolic compound with a diverse array of medicinal uses in the prevention of diabetes, cardiovascular diseases, cancer, and cerebral ischemia. Additionally, it exhibits antioxidant, antimicrobial, anti-inflammatory, neuroprotective, and hepatoprotective activities [73].

The accumulation of the organic acids citrate and malate in response to SeNP addition suggests a change in the tricarboxylic acid (TCA) cycle. Citrate and malate are key intermediates of the TCA cycle, essential for cellular energy production [40]. Moreover, The TCA cycle is playing a crucial role in ATP production and the provision of carbon skeletons for biosynthetic activities. Additionally, it contributes to carbon-nitrogen interaction and biotic stress response [90].

The presence of SeNP and Se at various concentrations leads to a reduction in the levels of certain aliphatic amino acids, such as arginine, glutamine, and tryptophan. These amino acids play crucial roles in the biosynthesis of secondary and bioactive metabolites. For example, tryptophan serves as a precursor for IAA, a key plant hormone involved in growth regulation [53]. Arginine is essential for the biosynthesis of polyamines, which are important for stress tolerance and cellular function. Meanwhile, glutamine is a precursor for the synthesis of γ-aminobutyric acid (GABA) and salicylic acid, both of which are involved in plant stress responses and defense mechanisms [16].

### KEGG pathway and enrichment analysis

Pathway and Enrichment analysis revealed that SeNP significantly changed amino acids as well as carbohydrate metabolism. The most significantly changed pathways include arginine biosynthesis, nitrogen metabolism, and galactose metabolism. The arginine biosynthesis pathway can be divided into two distinct stages. Initially, glutamate is converted into ornithine, which is then utilized for the subsequent production of arginine [83]. Arginine is a precursor for the synthesis of protein, polyamines and nitric oxide, which play a vital role in regulating plant growth, development, and stress responses [57, 84]. Arginine is known for its high nitrogen to carbon ratio among other proteinogenic amino acids and its effect on various processes in plants including photosynthesis and nutrient absorption [18]. Legume plants have a distinct nitrogen metabolism. Legumes have the ability to capture atmospheric nitrogen and enrich the soil with this essential nutrient. Nitrogen metabolism in legume nodules is regulated by many mechanisms that encompass the uptake, absorption, transport, and reutilization of nitrogen in different molecular forms [78]. White [82] reported that the nitrogen metabolism in plants is affected by Se uptake by the plant. The galactose metabolism pathway converts galactose into glucose-1-phosphate in several steps by the action of different enzymes; subsequently, glucose-1-phosphate can participate in glycolysis or the pentose phosphate pathway [36]. Galactose participates in cell wall biosynthesis [66], and is a precursor for biosynthesis of stachyose during stress conditions [21].

Pathway and enrichment analysis revealed that the addition of Se significantly changed glyoxylate and dicarboxylate metabolism; glycine-serine and threonine metabolism; and alanine, aspartate, and glutamate metabolism pathways. Glyoxylate and dicarboxylate metabolism has been reported in many studies as crucial in seed germination and early stages of growth in different plant species including wheat, Poplar and *Cyclobalanopsis gilva* seeds [63, 87, 89]. This pathway provides the plant with sufficient energy required for growth and development, and any limitation or inhibition in this pathway results in inhibition of the plant growth and development [13, 81]. Moreover, this metabolic pathway plays a crucial role in the plant’s response to environmental stress conditions like drought, salinity, and waterlogging [58, 60, 81].

The alanine-aspartate-glutamate metabolism pathway is important in plant growth and stress resistance. Alanine can be converted to pyruvate by alanine aminotransferases [35]. Alanine aminotransferase is linked to various biological processes in plants including the TCA cycle and glycolysis [68]. Aspartate and glutamate are critical because they are part of the biological pathways that incorporate fixed nitrogen into organic compounds. Glutamate acts as an amino group donor because it is highly abundant in legume nodule, and is incorporated in various biosynthetic processes [69]. Glutamate is a precursor for the biosynthesis of proline and glutathione [32, 79]. Aspartate is involved in the synthesis of other essential amino acids like lysine and methionine [10]. Aspartate is involved in the biosynthesis of the plant growth hormone, essential amino acids, and glycine betaine. Moreover, aspartate plays a crucial role in the tricarboxylic acid (TCA) cycle and is also involved in the biosynthesis of malate for the malic enzyme [10, 29]. The alteration of alanine-aspartate-glutamate in plants has been documented in other legume plants to heat stress [45].

The glycine, serine and threonine metabolism pathway plays a role in the growth and development of plants. Serine is involved in the synthesis of proteins, phospholipids, and other amino acids like cysteine and tryptophan [47]. Tryptophan, is the precursor of the phytohormone indole-3-acetic acid, a crucial controller of plant growth and development. Glycine is a precursor for the synthesis of stress related proteins [20]. Threonine is a precursor for the biosynthesis of isoleucine through the threonine deaminase pathway. This pathway is vital in balancing the synthesis of branched-chain amino acids and protein biosynthesis [44].

## Conclusion

The present study shows that SeNP at varying concentrations significantly influence the metabolome and biological pathways in *Phaseolus vulgaris*, leading to an increase in the accumulation of essential and bioactive metabolites, such as sulfur-containing amino acids, alkaloids and phenols. These findings underscore the potential of SeNP to enhance plant growth, defense, and stress tolerance, presenting a promising strategy for agricultural applications aimed at improving crop quality and resilience.

## Supplementary Information

Supplementary Material 1.

## Data Availability

The datasets generated and/or analysed during the current study are available in the figshare repository, 10.6084/m9.figshare.27855333.
