# Supplementary material for: Selenium nanoparticles enhance metabolic and nutritional profile in Phaseolus vulgaris: comparative metabolomic and pathway analysis with selenium selenate
Source: BMC Plant Biol. 2025 Jan 28;25:119. doi: 10.1186/s12870-025-06097-6 (PMC11773980; doi:10.1186/s12870-025-06097-6)
Supplement: Supplementary file 1 — Supplementary Material 1. [file 12870_2025_6097_MOESM1_ESM.docx]

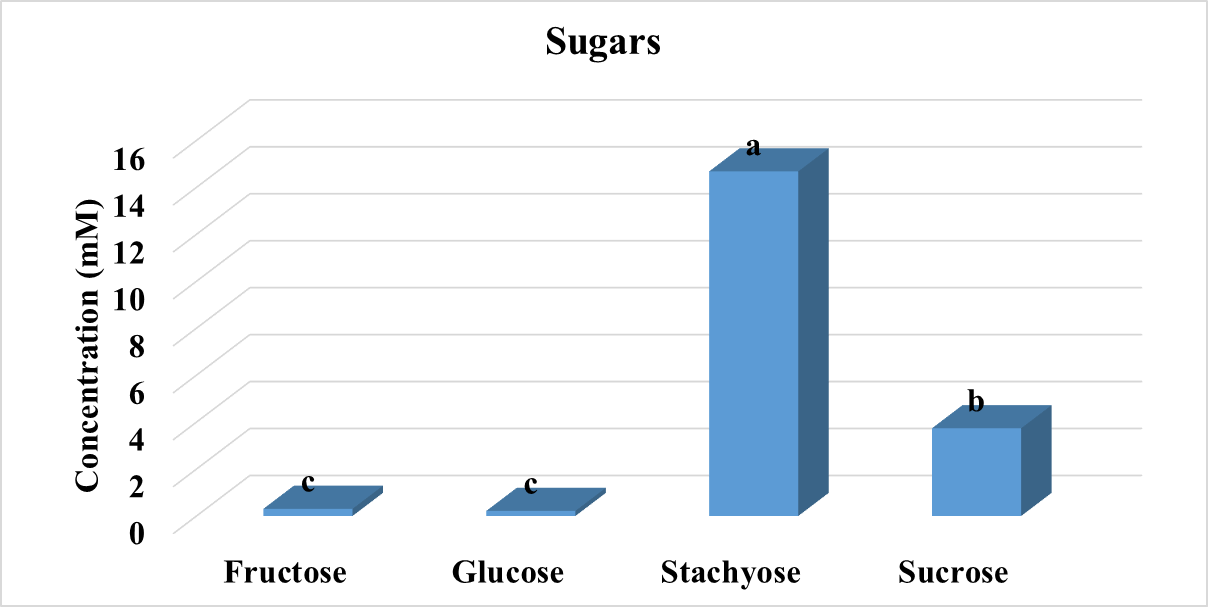


Figure 1: Concentrations of sugars identified in the polar extract of *Phaseolus vulgaris* seeds.


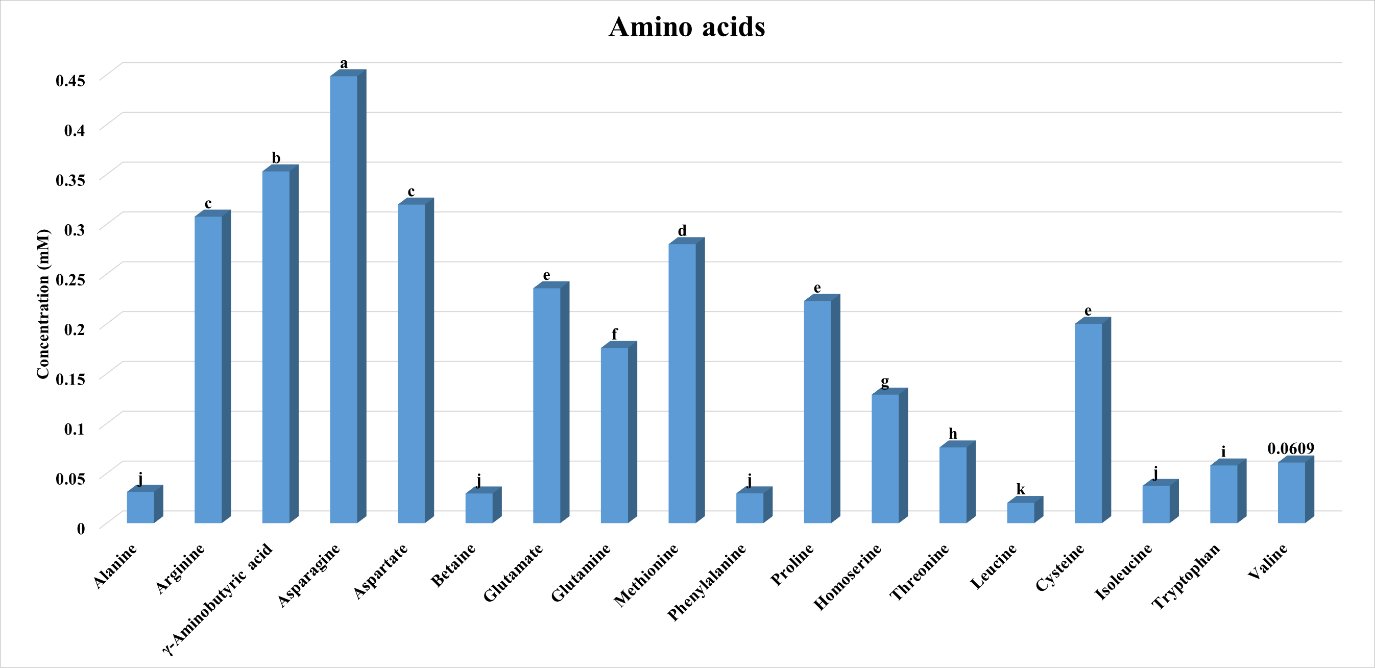


Figure 2: Concentrations of amino acids identified in the polar extract of *Phaseolus vulgaris* seeds.


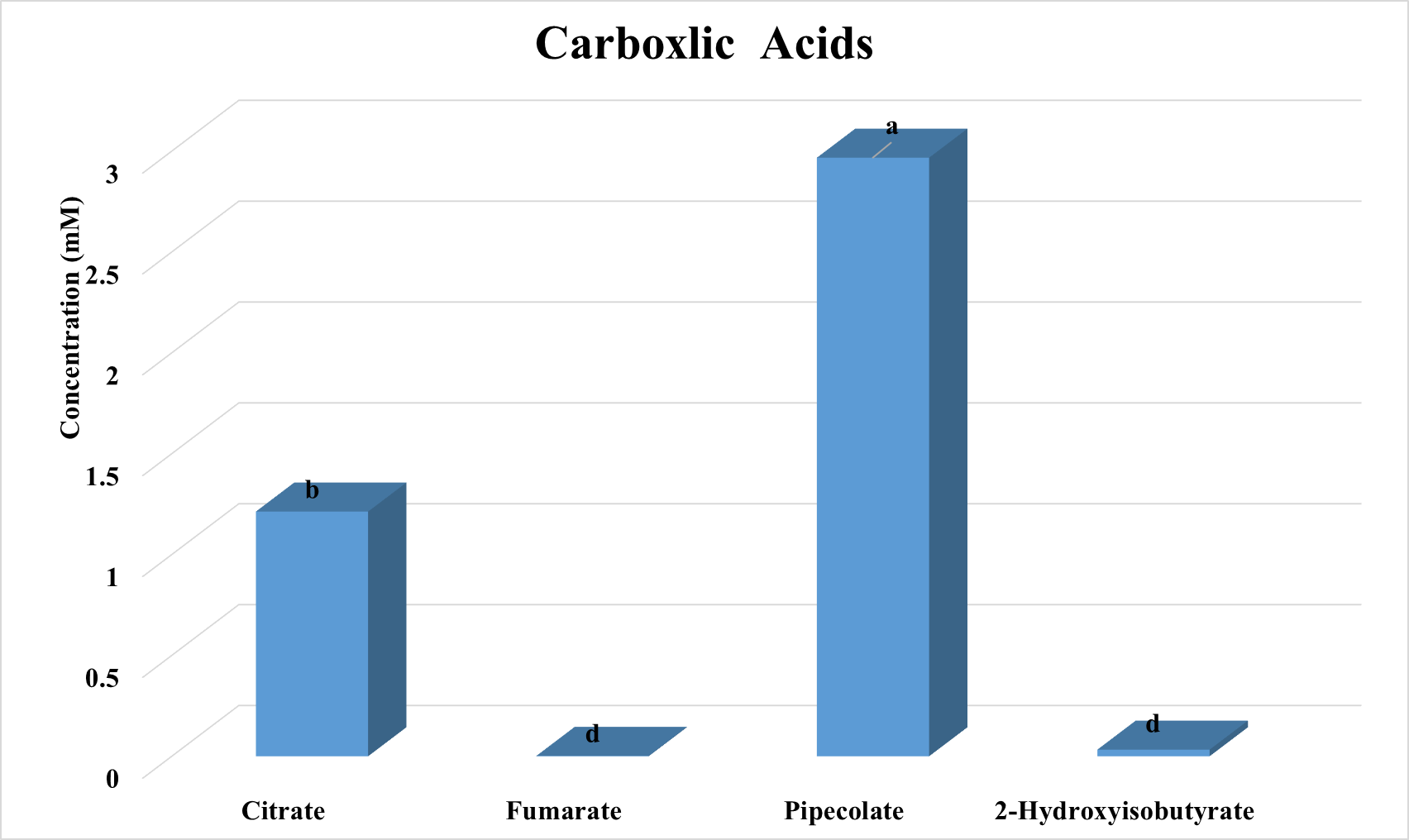


Figure 3: Concentrations of carboxlic acids identified in the polar extract of *Phaseolus vulgaris* seeds.


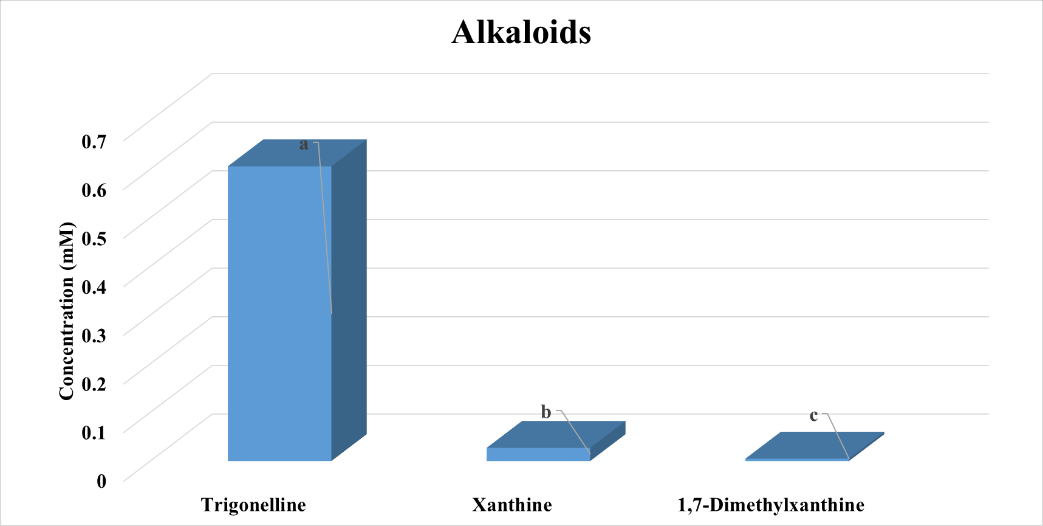


Figure 4: Concentrations of alkaloids identified in the polar extract of *Phaseolus vulgaris* seeds.

**Table 1:** Pathway analysis depicting significantly altered metabolic pathways in *Phaseolus vulgaris* seeds polar in response to **SeNP** addition. The table shows the detailed results from the pathway analysis. Since we are testing many pathways at the same time, the statistical p values from enrichment analysis are further adjusted for multiple testings. In particular, the “Total” is the total number of compounds in the pathway; the “Hits” is the actually matched number from the user uploaded data; the “Raw p” is the original p value calculated from the enrichment analysis; the Holm p is the p value adjusted by Holm-Bonferroni method; the “FDR p” is the p value adjusted using False Discovery Rate.

|  | **Pathway Name** | **Total** | **Expected** | **Hits** | **Raw p** | **Holm adjust** | **FDR** | **Impact** |
| --- | --- | --- | --- | --- | --- | --- | --- | --- |
| 1 | Arginine biosynthesis | 18 | 0.23077 | 4 | 5.29E-05 | 0.004809 | 0.004809 | 0.16991 |
| 2 | Nitrogen metabolism | 11 | 0.14103 | 3 | 0.000277 | 0.024902 | 0.008542 | 0 |
| 3 | Galactose metabolism | 27 | 0.34615 | 4 | 0.000282 | 0.025061 | 0.008542 | 0.07612 |
| 4 | Starch and sucrose metabolism | 22 | 0.28205 | 3 | 0.002361 | 0.20778 | 0.042972 | 0.40507 |
| 5 | Alanine, aspartate and glutamate metabolism | 22 | 0.28205 | 3 | 0.002361 | 0.20778 | 0.042972 | 0.64388 |
| 6 | Glyoxylate and dicarboxylate metabolism | 29 | 0.37179 | 3 | 0.005292 | 0.4551 | 0.080261 | 0.05591 |
| 7 | Glycine, serine and threonine metabolism | 33 | 0.42308 | 3 | 0.007649 | 0.65013 | 0.099431 | 0 |
| 8 | Cysteine and methionine metabolism | 47 | 0.60256 | 3 | 0.020284 | 1 | 0.23074 | 0.17481 |
| 9 | Valine, leucine and isoleucine biosynthesis | 22 | 0.28205 | 2 | 0.030887 | 1 | 0.29732 | 0 |
| 10 | Cyanoamino acid metabolism | 25 | 0.32051 | 2 | 0.039207 | 1 | 0.29732 | 0 |
| 11 | Pantothenate and CoA biosynthesis | 25 | 0.32051 | 2 | 0.039207 | 1 | 0.29732 | 0 |
| 12 | Tryptophan metabolism | 25 | 0.32051 | 2 | 0.039207 | 1 | 0.29732 | 0.5862 |
| 13 | Glutathione metabolism | 27 | 0.34615 | 2 | 0.045181 | 1 | 0.31388 | 0.05766 |
| 14 | Arginine and proline metabolism | 28 | 0.35897 | 2 | 0.048288 | 1 | 0.31388 | 0.09174 |
| 15 | Glucosinolate biosynthesis | 30 | 0.38462 | 2 | 0.054733 | 1 | 0.33205 | 0 |
| 16 | Valine, leucine and isoleucine degradation | 37 | 0.47436 | 2 | 0.079477 | 1 | 0.45202 | 0 |
| 17 | Pyrimidine metabolism | 41 | 0.52564 | 2 | 0.094946 | 1 | 0.49696 | 0.04269 |
| 18 | Monobactam biosynthesis | 8 | 0.10256 | 1 | 0.0983 | 1 | 0.49696 | 0 |
| 19 | Lysine biosynthesis | 9 | 0.11538 | 1 | 0.10992 | 1 | 0.52647 | 0 |
| 20 | Vitamin B6 metabolism | 12 | 0.15385 | 1 | 0.14395 | 1 | 0.65496 | 0 |
| 21 | Nicotinate and nicotinamide metabolism | 13 | 0.16667 | 1 | 0.15501 | 1 | 0.67171 | 0 |
| 22 | Sulfur metabolism | 15 | 0.19231 | 1 | 0.17674 | 1 | 0.73104 | 0.06077 |
| 23 | Butanoate metabolism | 17 | 0.21795 | 1 | 0.19793 | 1 | 0.75833 | 0 |
| 24 | beta-Alanine metabolism | 18 | 0.23077 | 1 | 0.20833 | 1 | 0.75833 | 0 |
| 25 | Fructose and mannose metabolism | 18 | 0.23077 | 1 | 0.20833 | 1 | 0.75833 | 0.06695 |
| 26 | Lysine degradation | 20 | 0.25641 | 1 | 0.22875 | 1 | 0.78033 | 0 |
| 27 | Carbon fixation in photosynthetic organisms | 21 | 0.26923 | 1 | 0.23878 | 1 | 0.78033 | 0 |
| 28 | Phenylalanine, tyrosine and tryptophan biosynthesis | 22 | 0.28205 | 1 | 0.24868 | 1 | 0.78033 | 0 |
| 29 | Thiamine metabolism | 22 | 0.28205 | 1 | 0.24868 | 1 | 0.78033 | 0 |
| 30 | Biosynthesis of various plant secondary metabolites | 24 | 0.30769 | 1 | 0.26811 | 1 | 0.81327 | 0 |
| 31 | Glycerophospholipid metabolism | 38 | 0.48718 | 1 | 0.3914 | 1 | 1 | 0.02493 |
| 32 | Porphyrin metabolism | 47 | 0.60256 | 1 | 0.45997 | 1 | 1 | 0 |
| 33 | Amino sugar and nucleotide sugar metabolism | 52 | 0.66667 | 1 | 0.49484 | 1 | 1 | 0 |
| 34 | Purine metabolism | 75 | 0.96154 | 1 | 0.62951 | 1 | 1 | 0 |

**Table 2**: Enrichment analysis depicting significantly altered metabolic pathways in *Phaseolus vulgaris* seeds polar in response to SeNP addition. The table shows the detailed results from the pathway analysis. Since we are testing many pathways at the same time, the statistical p values from enrichment analysis are further adjusted for multiple testings. In particular, the **Total** is the total number of compounds in the pathway; the **Hits** is the actually matched number from the user uploaded data; the **Raw p** is the original p value calculated from the enrichment analysis; the Holm p is the p value adjusted by Holm-Bonferroni method; the **FDR p** is the p value adjusted using False Discovery Rate.

|  | **Pathway Name** | **total** | **expected** | **hits** | **Raw p** | **Holm p** | **FDR** |
| --- | --- | --- | --- | --- | --- | --- | --- |
| 1 | Arginine biosynthesis | 14 | 0.155 | 4 | 9.56E-06 | 0.000765 | 0.000765 |
| 2 | Pantothenate and CoA biosynthesis | 20 | 0.221 | 3 | 0.00114 | 0.0899 | 0.0413 |
| 3 | Nitrogen metabolism | 6 | 0.0663 | 2 | 0.00168 | 0.131 | 0.0413 |
| 4 | Galactose metabolism | 27 | 0.298 | 3 | 0.00278 | 0.214 | 0.0413 |
| 5 | Valine, leucine and isoleucine biosynthesis | 8 | 0.0884 | 2 | 0.00309 | 0.235 | 0.0413 |
| 6 | Alanine, aspartate and glutamate metabolism | 28 | 0.309 | 3 | 0.00309 | 0.235 | 0.0413 |
| 7 | Glyoxylate and dicarboxylate metabolism | 31 | 0.342 | 3 | 0.00416 | 0.308 | 0.0475 |
| 8 | Histidine metabolism | 16 | 0.177 | 2 | 0.0126 | 0.919 | 0.126 |
| 9 | Starch and sucrose metabolism | 18 | 0.199 | 2 | 0.0158 | 1 | 0.141 |
| 10 | Glutathione metabolism | 28 | 0.309 | 2 | 0.0367 | 1 | 0.293 |
| 11 | Glycine, serine and threonine metabolism | 33 | 0.365 | 2 | 0.0496 | 1 | 0.331 |
| 12 | Cysteine and methionine metabolism | 33 | 0.365 | 2 | 0.0496 | 1 | 0.331 |
| 13 | Arginine and proline metabolism | 36 | 0.398 | 2 | 0.0581 | 1 | 0.353 |
| 14 | Pyrimidine metabolism | 39 | 0.431 | 2 | 0.067 | 1 | 0.353 |
| 15 | Valine, leucine and isoleucine degradation | 39 | 0.431 | 2 | 0.067 | 1 | 0.353 |
| 16 | Tryptophan metabolism | 41 | 0.453 | 2 | 0.0732 | 1 | 0.353 |
| 17 | Thiamine metabolism | 7 | 0.0773 | 1 | 0.0749 | 1 | 0.353 |
| 18 | Taurine and hypotaurine metabolism | 8 | 0.0884 | 1 | 0.0852 | 1 | 0.379 |
| 19 | Butanoate metabolism | 15 | 0.166 | 1 | 0.154 | 1 | 0.616 |
| 20 | Nicotinate and nicotinamide metabolism | 15 | 0.166 | 1 | 0.154 | 1 | 0.616 |
| 21 | Fructose and mannose metabolism | 20 | 0.221 | 1 | 0.2 | 1 | 0.761 |
| 22 | beta-Alanine metabolism | 21 | 0.232 | 1 | 0.209 | 1 | 0.761 |
| 23 | Porphyrin metabolism | 31 | 0.342 | 1 | 0.294 | 1 | 1 |
| 24 | Glycerophospholipid metabolism | 36 | 0.398 | 1 | 0.333 | 1 | 1 |
| 25 | Amino sugar and nucleotide sugar metabolism | 42 | 0.464 | 1 | 0.377 | 1 | 1 |
| 26 | Purine metabolism | 70 | 0.773 | 1 | 0.549 | 1 | 1 |

**Table 3:** Pathway analysis depicting significantly altered metabolic pathways in *Phaseolus vulgaris* seeds polar in response to Se addition. The table shows the detailed results from the pathway analysis. Since we are testing many pathways at the same time, the statistical p values from enrichment analysis are further adjusted for multiple testings. In particular, the **Total** is the total number of compounds in the pathway; the **Hits** is the actually matched number from the user uploaded data; the **Raw p** is the original p value calculated from the enrichment analysis; the Holm p is the p value adjusted by Holm-Bonferroni method; the **FDR p** is the p value adjusted using False Discovery Rate.

|  | **Pathway Name** | Total | Expected | Hits | Raw p | Holm adjust | FDR | Impact |
| --- | --- | --- | --- | --- | --- | --- | --- | --- |
| 1 | Glyoxylate and dicarboxylate metabolism | 29 | 0.41093 | 8 | 1.31E-09 | 1.19E-07 | 1.19E-07 | 0.13849 |
| 2 | Glycine, serine and threonine metabolism | 33 | 0.46761 | 6 | 3.25E-06 | 0.000293 | 0.000148 | 0.30115 |
| 3 | Alanine, aspartate and glutamate metabolism | 22 | 0.31174 | 5 | 7.76E-06 | 0.000691 | 0.000235 | 0.52158 |
| 4 | Arginine biosynthesis | 18 | 0.25506 | 4 | 8.04E-05 | 0.007073 | 0.001828 | 0.22136 |
| 5 | Citrate cycle (TCA cycle) | 20 | 0.2834 | 4 | 0.000125 | 0.010868 | 0.002274 | 0.21839 |
| 6 | Nitrogen metabolism | 11 | 0.15587 | 3 | 0.000377 | 0.032394 | 0.005713 | 0 |
| 7 | Sulfur metabolism | 15 | 0.21255 | 3 | 0.001001 | 0.085114 | 0.013017 | 0.09392 |
| 8 | Pyruvate metabolism | 23 | 0.32591 | 3 | 0.003622 | 0.30427 | 0.041203 | 0.23929 |
| 9 | Vitamin B6 metabolism | 12 | 0.17004 | 2 | 0.011592 | 0.96214 | 0.11721 | 0.02381 |
| 10 | Butanoate metabolism | 17 | 0.24089 | 2 | 0.022889 | 1 | 0.20829 | 0 |
| 11 | Carbon fixation in photosynthetic organisms | 21 | 0.29757 | 2 | 0.03416 | 1 | 0.28254 | 0.05879 |
| 12 | Valine, leucine and isoleucine biosynthesis | 22 | 0.31174 | 2 | 0.037258 | 1 | 0.28254 | 0 |
| 13 | D-Amino acid metabolism | 7 | 0.09919 | 1 | 0.095257 | 1 | 0.64386 | 0 |
| 14 | Glycerophospholipid metabolism | 38 | 0.53846 | 2 | 0.099055 | 1 | 0.64386 | 0.02862 |
| 15 | Pyrimidine metabolism | 41 | 0.58097 | 2 | 0.11267 | 1 | 0.68353 | 0.04269 |
| 16 | Selenocompound metabolism | 13 | 0.18421 | 1 | 0.16997 | 1 | 0.96668 | 0 |
| 17 | Tyrosine metabolism | 18 | 0.25506 | 1 | 0.2277 | 1 | 1 | 0.07027 |
| 18 | Propanoate metabolism | 19 | 0.26923 | 1 | 0.23878 | 1 | 1 | 0 |
| 19 | Phenylalanine, tyrosine and tryptophan biosynthesis | 22 | 0.31174 | 1 | 0.27111 | 1 | 1 | 0 |
| 20 | Purine metabolism | 75 | 1.0628 | 2 | 0.28787 | 1 | 1 | 0.00637 |
| 21 | Cyanoamino acid metabolism | 25 | 0.35425 | 1 | 0.30213 | 1 | 1 | 0 |
| 22 | Tryptophan metabolism | 25 | 0.35425 | 1 | 0.30213 | 1 | 1 | 0.17241 |
| 23 | Glycolysis / Gluconeogenesis | 26 | 0.36842 | 1 | 0.31219 | 1 | 1 | 0.00147 |
| 24 | Sphingolipid metabolism | 27 | 0.38259 | 1 | 0.32211 | 1 | 1 | 0 |
| 25 | Glutathione metabolism | 27 | 0.38259 | 1 | 0.32211 | 1 | 1 | 0.05016 |
| 26 | Galactose metabolism | 27 | 0.38259 | 1 | 0.32211 | 1 | 1 | 0.30161 |
| 27 | Arginine and proline metabolism | 28 | 0.39676 | 1 | 0.3319 | 1 | 1 | 0 |
| 28 | Glucosinolate biosynthesis | 30 | 0.4251 | 1 | 0.35106 | 1 | 1 | 0 |
| 29 | Valine, leucine and isoleucine degradation | 37 | 0.52429 | 1 | 0.4141 | 1 | 1 | 0 |
| 30 | Cysteine and methionine metabolism | 47 | 0.66599 | 1 | 0.49413 | 1 | 1 | 0 |
| 31 | Porphyrin metabolism | 47 | 0.66599 | 1 | 0.49413 | 1 | 1 | 0 |

**Table 4**: Enrichment analysis depicting significantly altered metabolic pathways in *Phaseolus vulgaris* seeds polar in response to Se addition. The table shows the detailed results from the pathway analysis. Since we are testing many pathways at the same time, the statistical p values from enrichment analysis are further adjusted for multiple testings. In particular, the **Total** is the total number of compounds in the pathway; the **Hits** is the actually matched number from the user uploaded data; the **Raw p** is the original p value calculated from the enrichment analysis; the Holm p is the p value adjusted by Holm-Bonferroni method; the **FDR p** is the p value adjusted using False Discovery.

|  | **Pathway Name** | total | expected | hits | Raw p | Holm p | FDR |
| --- | --- | --- | --- | --- | --- | --- | --- |
| 1 | Glyoxylate and dicarboxylate metabolism | 31 | 0.443 | 7 | 9.10E-08 | 7.28E-06 | 7.28E-06 |
| 2 | Alanine, aspartate and glutamate metabolism | 28 | 0.4 | 6 | 1.26E-06 | 9.96E-05 | 5.04E-05 |
| 3 | Arginine biosynthesis | 14 | 0.2 | 4 | 2.86E-05 | 0.00223 | 0.000763 |
| 4 | Glycine, serine and threonine metabolism | 33 | 0.472 | 5 | 6.74E-05 | 0.00519 | 0.00135 |
| 5 | Citrate cycle (TCA cycle) | 20 | 0.286 | 4 | 0.000131 | 0.00995 | 0.00209 |
| 6 | Nitrogen metabolism | 6 | 0.0858 | 2 | 0.00283 | 0.212 | 0.0377 |
| 7 | Pyruvate metabolism | 23 | 0.329 | 3 | 0.00373 | 0.276 | 0.0427 |
| 8 | Valine, leucine and isoleucine biosynthesis | 8 | 0.114 | 2 | 0.00519 | 0.379 | 0.0519 |
| 9 | Butanoate metabolism | 15 | 0.214 | 2 | 0.0183 | 1 | 0.163 |
| 10 | Histidine metabolism | 16 | 0.229 | 2 | 0.0207 | 1 | 0.166 |
| 11 | Pyrimidine metabolism | 39 | 0.558 | 2 | 0.105 | 1 | 0.765 |
| 12 | Vitamin B6 metabolism | 9 | 0.129 | 1 | 0.122 | 1 | 0.812 |
| 13 | D-Amino acid metabolism | 15 | 0.214 | 1 | 0.195 | 1 | 1 |
| 14 | Selenocompound metabolism | 20 | 0.286 | 1 | 0.252 | 1 | 1 |
| 15 | Propanoate metabolism | 21 | 0.3 | 1 | 0.262 | 1 | 1 |
| 16 | Purine metabolism | 70 | 1 | 2 | 0.264 | 1 | 1 |
| 17 | Glycolysis / Gluconeogenesis | 26 | 0.372 | 1 | 0.314 | 1 | 1 |
| 18 | Galactose metabolism | 27 | 0.386 | 1 | 0.324 | 1 | 1 |
| 19 | Glutathione metabolism | 28 | 0.4 | 1 | 0.334 | 1 | 1 |
| 20 | Porphyrin metabolism | 31 | 0.443 | 1 | 0.363 | 1 | 1 |
| 21 | Sphingolipid metabolism | 32 | 0.457 | 1 | 0.372 | 1 | 1 |
| 22 | Cysteine and methionine metabolism | 33 | 0.472 | 1 | 0.381 | 1 | 1 |
| 23 | Arginine and proline metabolism | 36 | 0.515 | 1 | 0.408 | 1 | 1 |
| 24 | Glycerophospholipid metabolism | 36 | 0.515 | 1 | 0.408 | 1 | 1 |
| 25 | Valine, leucine and isoleucine degradation | 39 | 0.558 | 1 | 0.434 | 1 | 1 |
| 26 | Tryptophan metabolism | 41 | 0.586 | 1 | 0.45 | 1 | 1 |
| 27 | Tyrosine metabolism | 42 | 0.6 | 1 | 0.458 | 1 | 1 |
